# Higher -Order Assembly of BRCC36–KIAA0157 Is Required for DUB Activity and Biological Function

**DOI:** 10.1016/j.molcel.2015.07.028

**Published:** 2015-09-03

**Authors:** Elton Zeqiraj, Lei Tian, Christopher A. Piggott, Monica C. Pillon, Nicole M. Duffy, Derek F. Ceccarelli, Alexander F. A. Keszei, Kristina Lorenzen, Igor Kurinov, Stephen Orlicky, Gerald D. Gish, Albert J.R. Heck, Alba Guarné, Roger A. Greenberg, Frank Sicheri

**Affiliations:** 1 Lunenfeld-Tanenbaum Research Institute, Mount Sinai Hospital, 600 University Avenue, Room 1090, Toronto, Ontario, Canada, M5G 1X5; 2 Departments of Cancer Biology and Pathology, Abramson Family Cancer Research Institute, Basser Research Center for BRCA, Perelman School of Medicine, University of Pennsylvania, 421 Curie Blvd, Philadelphia, PA 19104-6160; 3 Department of Biochemistry and Biomedical Sciences, McMaster University, Hamilton, ON L8S 4K1, Canada; 4 Departments of Biochemistry and Molecular Genetics, University of Toronto, Toronto, Canada M5S 1A8; 5 Department of Chemistry and Chemical Biology, Cornell University, Argonne, IL 60439, USA; 6 Bijvoet Center for Biomolecular Research and Utrecht Institute for Pharmaceutical Science, Utrecht University, Padualaan 8, 3584 CH, Utrecht, The Netherlands

## Abstract

BRCC36 is a Zn^2+^ dependent deubiquitinating enzyme (DUB) that hydrolyzes lysine-63-linked ubiquitin chains as part of distinct macromolecular complexes that participate in either interferon signaling or DNA-damage recognition. The MPN^+^ domain protein BRCC36 associates with pseudo-DUB MPN^−^ proteins KIAA0157 or Abraxas, which are essential for BRCC36 enzymatic activity. To understand the basis for BRCC36 regulation, we have solved the structure of an active BRCC36-KIAA0157 heterodimer and an inactive BRCC36 homodimer. Structural and functional characterizations show how BRCC36 is switched to an active conformation by contacts with KIAA0157. Higher order association of BRCC36 and KIAA0157 into a dimer of heterodimers (super dimers) was required for DUB activity and interaction with targeting proteins SHMT2 and RAP80. These data provide the first explanation of how an inactive pseudo DUB allosterically activates a cognate DUB partner, and implicates super dimerization as a new regulatory mechanism underlying BRCC36 DUB activity, subcellular localization, and biological function.

## INTRODUCTION

Ubiquitin mediated signal transduction depends on a balance between tightly regulated ubiquitin ligase and deubiquitinating enzyme (DUB) activities (Komander and Rape, 2012; Komander et al., 2009). Additional layers of control occur at the level of ubiquitin binding domain containing proteins that influence the localization of ubiquitin-directed enzymatic activity to specific subcellular compartments. Polyubiquitin chains linked through lysine 63 of ubiquitin (K63-Ub) are synthesized by specific E2 ubiquitin ligase enzyme pairs (Ubc13–Uev1A) and can be selectively recognized by ubiquitin binding domains (e.g. tandem UIMs in RAP80 and NZF domains in TAB2 (Husnjak and Dikic, 2012; Sato et al., 2009; Sims and Cohen, 2009; Sobhian et al., 2007), and hydrolyzed in a linkage selective manner by specific deubiquitinating enzymes (Eletr and Wilkinson, 2014). K63-Ub chains are utilized in signaling events in both the cytoplasm and nucleus, and do not target proteins for degradation by the proteasome.

BRCC36 is the catalytic subunit responsible for the majority of K63-Ub specific DUB activity in the cytoplasm and in the nucleus as part of two distinct macromolecular assemblies characterized by the presence of either of the MPN^−^ pseudo DUB proteins KIAA0157 or Abraxas (Cooper et al., 2009; Dong et al., 2003; Sobhian et al., 2007). MERIT40 and BRCC45 are common subunits to both complexes. The Abraxas complex (ARISC) functions in DNA repair and breast cancer suppression, while the BRCC36 isopeptidase complex (BRISC) promotes interferon dependent responses by deubiquitinating and stabilizing the type I interferon receptor, IFNAR1. Both BRCC36 complexes demonstrate tightly regulated DUB activity involving protein-protein interactions with their pseudo DUB subunits and with targeting proteins SHMT2 or RAP80. The latter two target the complexes to K63-Ub chains that are synthesized locally in response to cytokine receptor activation for BRISC or DNA double-strand breaks (DSBs) for ARISC (Kim et al., 2007; Sobhian et al., 2007; Wang et al., 2007; Zheng et al., 2013).

BRCC36 consists of a functional metalloprotease JAMM/MPN^+^ domain followed by a predicted coiled coil region implicated in protein-protein interactions. MPN^+^ domain DUBs share a common fold architecture and catalytic mechanism for peptide bond cleavage involving coordination of a Zn^2+^ ion by two histidines and one aspartate, an active site glutamic acid that delivers a water molecule for hydrolysis of isopeptide bonds, and an active site serine residue thought to stabilize the tetrahedral transition state intermediate in a similar mechanism to that of Zn metalloproteases subtilisin and thermolysin (Ambroggio et al., 2003; Tran et al., 2003). MPN^+^ DUBs are implicated in cleaving ubiquitin chains from unfolded proteins (e.g. RPN11 subunit within the proteasome), while others show specificity for hydrolysis of the ubiquitin like protein Nedd8 (e.g. CSN5 cleaving Nedd8 from Cullins), and finally, several are highly selective for particular chain linkages (*e.g.* AMSH and BRCC36). The archetype family member AMSH-LP cleaves K63 linked ubiquitin chains and represents the only member for which a structural basis for chain recognition has been determined (Sato et al., 2008).

MPN^+^ domain DUBs can act in isolation, as exemplified by AMSH-LP, or in macromolecular assemblies to cleave poly ubiquitin chains of varied linkage types. In protein complexes, an MPN^+^ domain can associate with pseudo MPN domains (MPN^−^) for purposes that are not well understood. MPN^−^ domains are recognizable by the absence of essential Zn^2+^-coordinating residues that are required for catalytic function. Established MPN^+^–MPN^−^ domain pairings include CSN5 and CSN6 in the COP9 signalosome and RPN11 and RPN8 in the proteasome. While the mode of MPN^+^–MPN^−^ interaction is conserved between these two systems (Deshaies, 2014; Lingaraju et al., 2014; Pathare et al., 2014; Worden et al., 2014), no precise function has been ascribed to either MPN^−^ domain, and neither structure has been probed deeply for insights into how the MPN^−^ domain might support MPN^+^ domain function.

The importance of the MPN^−^ subunit in BRISC and ARISC minimally serves a two-fold purpose, first to support DUB activity and second to mediate protein–protein interactions. KIAA0157 and Abraxas are required for the catalytic function of BRCC36 in both complexes, and enable association of two additional shared subunits (BRCC45 and MERIT40) to each holoenzyme. Moreover, BRCC36 interactions with KIAA0157 or Abraxas are necessary for association with the holoenzyme specific adapter subunits, SHMT2 and RAP80, which target their respective DUB complex to sites of K63-Ub synthesis.

In this study we sought to address, (1) the general architecture of active BRCC36 DUB complexes, and (2) how subunit assembly affects enzyme function. Towards this end, we have characterized the binding architecture of BRISC and ARISC complexes and solved the X-ray crystal structures of the minimally active BRCC36–KIAA0157 heterodimer subcomplex, and an inactive BRCC36-BRCC36 homodimer complex. These studies revealed that formation of an unanticipated higher order super dimer is essential for DUB activity and for protein interactions necessary for the subcellular localization and biological function of each BRCC36 complex.

## RESULTS

### Assembly overview of the BRISC complex

To probe the assembly architecture of BRISC and ARISC complexes for downstream structure and function analyses, we surveyed the expression of complete holoenzyme complexes and subcomplexes in insect cells from a variety of metazoan orthologues, including H. sapiens, X. laevis, G. gallus, D. rerio, C. floridanus and *A. thaliana* (see Table S1 for a summary of gene names and accession numbers). Using the MultiBac expression system (Fitzgerald et al., 2006), we expressed and purified DrBRCC36 and CfBRCC36 in isolation, *C. floridanus* BRCC36–KIAA0157, BRCC36–KIAA0157–BRCC45 and BRCC45–MERIT40 subcomplexes, and fully intact *C. floridanus, D. rerio* and *H. sapiens* BRISC and the *H. Sapiens* ARISC (BRCC36–Abraxas–BRCC45–MERIT40) complexes at preparative levels, either as full length proteins or as trimmed deletion mutants optimized for crystallization Figure S1).

**Figure 1:**
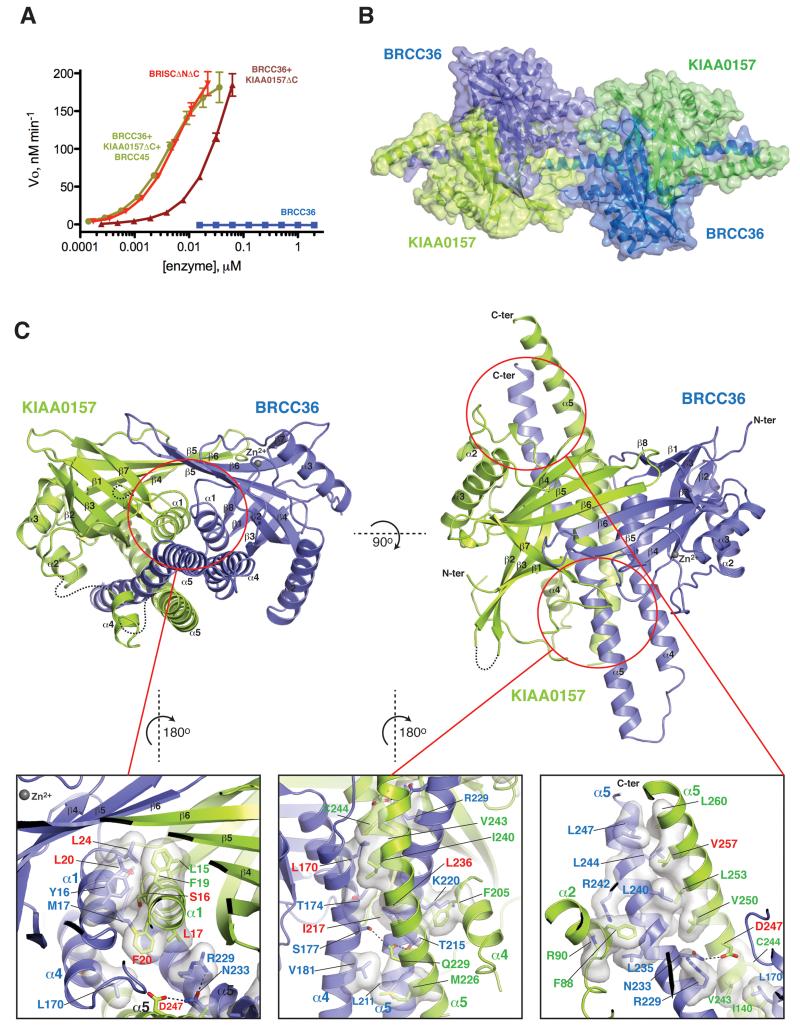
Structure of the BRCC36–KIAA0157 complex **A)** Cleavage activity of CfBRCC36 and CfBRCC36-containing complexes towards an internally quenched K63-diUb fluorogenic substrate. Results ± SEM are the average of three independent experiments carried out in duplicate. **B)** Structure of the CfBRCC36–KIAA0157ΔC complex. Contents of the asymmetric unit revealed two CfBRCC36–KIAA0157ΔC complexes forming a dimer of heterodimers (super dimer). **C)** Ribbon representation of the CfBRCC36–KIAA0157ΔC heterodimer. Zoom in panels show a detailed view of interacting residues. Disordered regions are shown as dashed lines. Interacting residues analyzed by mutation are labeled in red. See also Figures S1-S4 and S6.

CfBRCC36 alone displayed no detectable cleavage activity against a K63-linked diUb fluorigenic substrate in vitro (Figure 1A). However when complexed with CfKIAA0157, the protein displayed robust DUB activity that was further enhanced by the presence of CfBRCC45. Further addition of CfMERIT40 had no further effect on DUB activity. A similar trend of DUB activation was observed for zebrafish BRCC36 (Figure S1A) consistent with prior studies on human BRCC36 (Cooper et al., 2010; Feng et al., 2010; Patterson-Fortin et al., 2010). These results support the notion that the underlying mechanism of BRCC36 regulation by subunit assembly displayed by human, ant, and zebrafish proteins, is conserved throughout evolution.

Size exclusion chromatography coupled to multi-angle light scattering analysis (SEC-MALS) of the *C. floridanus* BRISC complex (Figure S1B), revealed a molecular weight of 256 kDa suggestive of a 2:2:2:2 stoichiometry (a super dimer). Molecular weights corresponding to super dimers were also observed for human BRISC and ARISC complexes (Figure S1C) and for the zebrafish BRISC complex (Figure S1D). To identify the subunit(s) responsible for super dimerization we subjected the following subcomplexes to SEC-MALS analysis. The Cf and Dr BRCC36–KIAA0157 binary complexes (131 and 130 kDa respectively), CfBRCC36–KIAA0157–BRCC45 triple complex (188 kDa) but not the CfBRCC45–MERIT40 binary complex (72 kDa) showed evidence of tight super dimerization (Figure S1B, D). We were unable to produce CfKIAA0157 and CfBRCC45 in isolation, and while CfBRCC36 was expressed at appreciable levels, it eluted as an aggregate (Figure S1B). DrBRC36 in isolation however, was well behaved in solution and displayed only a weak ability to form dimers (Figure S1E). These results (summarized in Figure S1F, G) were consistent with subunit assembly and stoichiometry models obtained using mass spectrometry measurements on HsBRISC under native conditions (Figure S2A-D).

Collectively, these data demonstrated several obligate protein folding relationships between MPN^−^ proteins and BRCC36, between BRCC45 and MERIT40 and between BRCC45 and BRCC36/KIAA0157, suggestive of direct binding interactions between subunits. They also indicated that both BRISC and ARISC adopt a tight higher order dimer configuration through the core BRCC36–KIAA0157 and BRCC36–Abraxas subcomplexes.

### Determination of a BRCC36–KIAA0157 co-structure

We pursued crystallization of BRCC36–KIAA0157 subcomplexes from different species (H. sapiens, G. gallus, X. tropicalis, D. rerio, C. floridanus and *A. thaliana*) to determine the structural basis for (1) how KIAA0157 supports the catalytic function of BRCC36, and (2) how super dimerization of the minimally active BRCC36–KIAA0157 heterodimer is mediated. Only CfBRCC36–KIAA0157 yielded initial crystals, but these diffracted poorly. Optimization of the CfKIAA0157 construct boundaries by deletion of poorly conserved C-terminal residues 290 to 471 (CfKIAA0157ΔC) yielded crystals in complex with full length CfBRCC36 (residues 1-253) that diffracted to 2.55 Å resolution.

We solved the CfBRCC36–KIAA0157ΔC crystal structure using the seleno-methionine-SAD method (See Methods for seleno-methionine protein production and crystallographic details). Following manual rebuilding and refinement, two highly interdigitated CfBRCC36–KIAA0157ΔC heterodimers were apparent in the asymmetric unit, arranged in the suspected super dimer configuration (Figure 1B). The final model displayed good crystallographic and geometric statistics (*R_work_*/*R_free_* = 0.189/0.240; see Table 1 for data collection and refinement statistics). Unbiased electron density maps are shown in Figure S2E-G and structure-based sequence alignments of BRCC36 and KIAA0157ΔC are shown in Figure S3.

### Structure of the BRCC36–KIAA0157 heterodimer

The structure of each protomer of CfBRCC36 and CfKIAA0157 consisted of a canonical MPN domain (residues 1–165 in BRCC36 comprising an eight strand β-sheet flanked by three α-helices and residues 1–180 in KIAA0157 comprising a seven strand β-sheet flanked by three α-helices), followed by two non-canonical helices α4 and α5 within the predicted coiled coil regions (Figure 1 and Figure S3). CfBRCC36 and CfKIAA0157ΔC protomers heterodimerized through a large buried surface area (SA) of 8112 Å^2^. The MPN domains of each associated in a pseudo two-fold manner centered on helices α1 (Figure 1C), resulting in a unified β-sheet and “fused” MPN^+^–MPN^−^ superstructure (buried SA = 2902 Å^2^). Non-canonical helices α4 and α5 of CfBRCC36 interacted with helices α4 and α5 of CfKIAA0157 in the form of a coiled coil helical bundle (CCHB) (Figure 1C). The helical bundle was tethered against the fused MPN domains of BRCC36 and KIAA0157 via a mixture of hydrophobic and hydrophilic interactions (Figure 1C).

The contact surfaces between CfBRCC36 and CfKIAA0157 protomers were highly hydrophobic in nature, which may account for our inability to produce CfKIAA0157 alone and the tendency of CfBRCC36 to form aggregates when expressed in isolation (Figure S1B). Three large networks of hydrophobic interactions were apparent, involving, firstly the MPN core domains (Leu15, Ser16, Leu17 and Phe20 from KIAA0157 α1 helix, and Tyr16, Met17, Leu20 and Leu24 from BRCC36 α1 helix), secondly, the N-terminal end of the CCHB (Leu170, Thr174 and Val181 from CfBRCC36 helix α4, and Met226, Leu236, Ile240, Val243 and Cys244 from CfKIAA0157 helix α5) and lastly, the C-terminal end of the CCHB (Arg229, Leu240, Leu244 and Leu247 of CfBRCC36 helix α5 and Val243, Val250, Leu253, Leu257 and Leu260 of CfKIAA0157 helix α5). A limited number of H-bonding interactions were also observed between Asp247 and Gln229 of KIAA0157 with Asn233, Ser177 and Thr215 of BRCC36 (Figure 1C).

**Figure 2:**
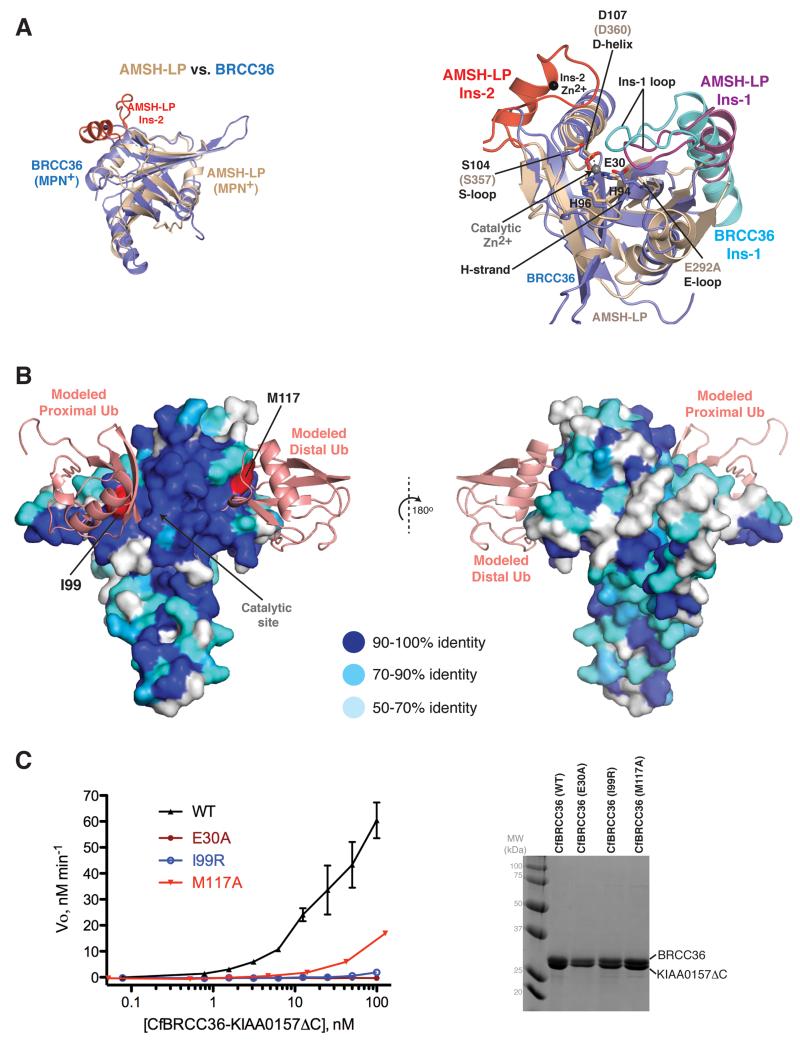
Catalytic and substrate binding infrastructure of the BRCC36–KIAA0157 complex **A)** Left, comparison of the CfBRCC36 MPN domain with AMSH-LP (PDBID 2ZNV). Right, zoom in view of the superimposed active site regions. All superpositions were performed using Coot and coiled coil regions were omitted for simplicity. **B)** Surface representation of CfBRCC36 structure colored in shades of blue reflecting sequence conservation across metazoan species (see sequence alignment in Figure S3). The binding of K63-linked diUb substrate was modeled by superimposing the AMSH-LP–diUb co-structure (PDBID 2ZNV) on the CfBRCC36 structure using the program Coot. Invariant residues predicted to interact with distal and proximal ubiquitins and selected for mutagenesis are colored red. **C)** Cleavage activity of WT CfBRCC36–KIAA0157ΔC and the indicated CfBRCC36 mutants towards an internally quenched K63-diUb fluorogenic substrate (left). Results ± SEM are the average of three independent experiments carried out in duplicate. Coomassie-stained SDS-PAGE analysis of the indicated complexes (right). See also Figures S3 and S4.

The heterodimer configuration of BRCC36–KIAA0157 contrasted with the archetypal member AMSH-LP, which exists as a simple MPN^+^ domain monomer (Figure 2A left). It was also surprisingly distinct from all known MPN^+^–MPN^−^ dimer configurations including the biologically relevant configuration shared by CSN5–CSN6 (buried SA = 7697 Å^2^; 4550 Å^2^ for MPN domains only) and RPN11–RPN8 (buried SA = 4054 Å^2^ for MPN domains only) (Figure S4A, B left panels), and the homodimer configurations of unknown relevance displayed by other MPN proteins (Figure S4C).

### Structure of the BRCC36–KIAA0157 heterodimer is functionally relevant in cells

**Figure 3:**
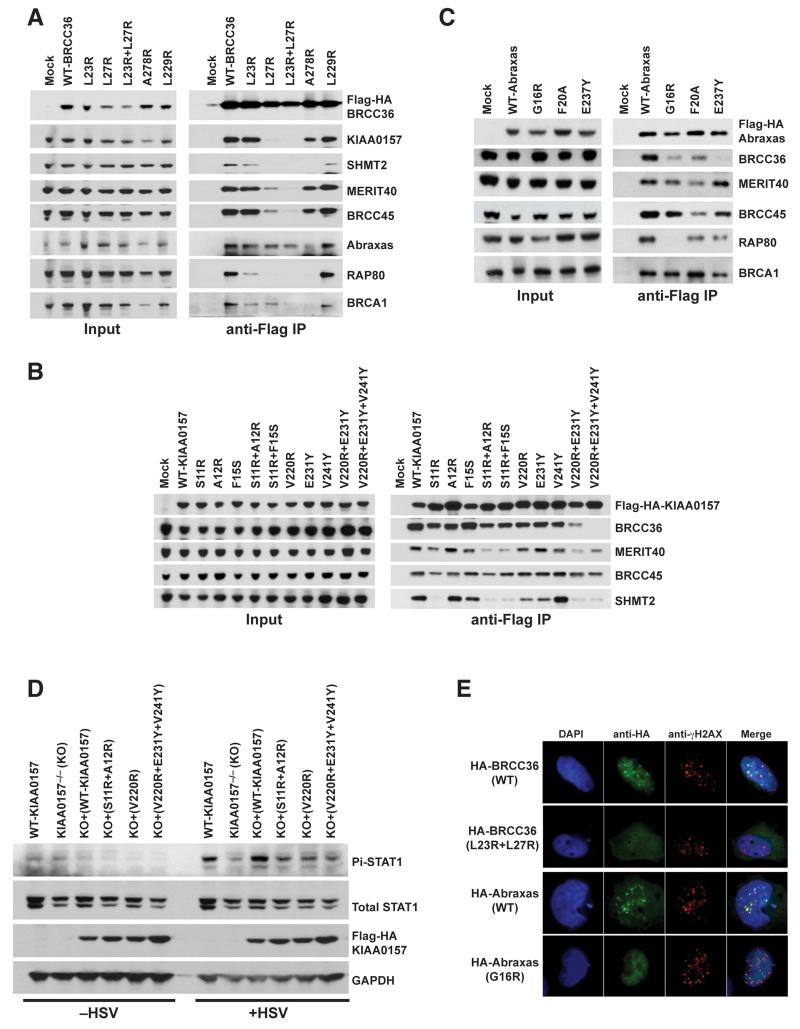
BRCC36–KIAA0157 interaction is important for signaling in cells **A)** Immunoprecipitation (IP) was performed using anti-Flag antibody in 293T cells that had been transiently transfected with Flag-HA epitope tagged BRCC36 wildtype (WT) or mutants. Immunoblot was performed using antibodies to the indicated BRISC and ARISC components. **B & C)** Flag-IP was performed for Flag-HA-KIAA0157 or Abraxas in transiently transfected 293T cells as in (**A**). Immunoblot was performed for selected members of the BRISC or ARISC as indicated to determine the impact of the various mutants tested on protein-protein interactions **D)** KIAA0157^−/−^ MEFs and KIAA0157^−/−^ MEFs reconstituted with WT or the indicated mutants were infected with Herpes Simplex Virus (HSV) deleted for the lytic phase gene ICP0. Interferon receptor dependent signal transduction response was assessed by immunoblot for STAT1 phosphorylated at Y701 (Pi-STAT1). **E)** Dual color immunofluoresence using antibodies against HA and γH2AX was performed at 6 hours after 10 Gy IR in MEFs that stably expressed either Flag-HA-BRCC36 or Flag-HA-Abraxas. Co-localization of WT BRCC36 or WT Abraxas with γH2AX indicates successful recruitment to DNA double-strand breaks. BRCC36 (L23R + L27R) or Abraxas (G16R) mutants failed to show foci formation and colocalization with γH2AX. Quantification is shown in Figure S5. See also Figure S4 and S5.

To validate the BRCC36–KIAA0157 heterodimer as being relevant in cells, we mutated direct contact residues and assessed interaction function of human BRCC36 and KIAA0157 by co-immunoprecipitation (see structure-based sequence alignments in Figure S3 and Table S2 for a list of equivalent residues mutated). The MPN^+^ mutation Leu27Arg alone or in combination with Leu23Arg in BRCC36 disrupted binding to KIAA0157, and in addition to MERIT40, BRCC45, SHMT2, RAP80, BRCA1 and to a lesser extent Abraxas (Figure 3A). In the reciprocal analysis, the MPN^−^ domain mutation Ser11Arg in KIAA0157 alone or in combination with Ala12Arg or Phe15Ser also reduced interaction with BRCC36 and additionally with MERIT40 and SHMT2 although to different extents (Figure 3B). The KIAA0157 CCHB double (Val220Arg + Glu231Tyr) and triple (Val220Arg + Glu231Tyr + Val241Tyr) mutations showed more potently reduced interaction with BRCC36, MERIT40 and SHMT2 (Figure 3B). Mutagenesis of the MPN^−^ domain of Abraxas, including Gly16Arg (analogous to Ser11 in KIAA0157) disrupted interaction with BRCC36 and RAP80, leaving interactions with BRCC45 and MERIT40 largely intact. A second MPN^−^ domain mutation, Phe20Ala, modestly reduced binding to BRCC36, and also to MERIT40, BRCC45 and RAP80 (Figure 3C). Lastly, the CCHB mutation Glu237Tyr in Abraxas potently disrupted binding to BRCC36 with only a modest effect on RAP80 interaction (Figure 3C). These observations support the notion that the crystal structure of the BRCC36-KIAA0157 heterodimer is reflective of both BRCC36-KIAA0157 and the BRCC36-Abraxas interactions in cells, and that perturbation of heterodimerization has far reaching consequences on the assembly of other subunits.

To assess the consequence of perturbing BRCC36–KIAA0157 heterodimerization on signaling function, we monitored STAT1 phosphorylation at Tyr701 in response to Herpes Simplex Virus (HSV) infection in KIAA0157^−/−^ mouse embryonic fibroblasts that had been reconstituted with either WT human KIAA0157 or the aforementioned KIAA0157 mutants. HSV lacking the lytic phase gene ICP0 produces robust interferon dependent JAK-STAT phosphorylation several hours following infection of human or mouse cells. KIAA0157^−/−^ MEFs showed reduced STAT1 phosphorylation (Figure 3D), consistent with impaired type I interferon signaling (Zheng et al., 2013). STAT1 phosphorylation was restored with introduction of WT KIAA0157, but showed reduced activation in KIAA0157^−/−^ cells expressing the MPN^−^ (Ser11Arg + Ala12Arg) mutant, or the CCHB single (Val220Arg) or triple (Val220Arg + Glu231Tyr + Val241Tyr) mutant. Similarly, BRCC36 MPN^+^ domain double mutant (Leu23Arg + Leu27Arg) was impaired for IR–induced foci formation as was Abraxas MPN^−^ mutant (Gly16Arg) (Figure 3E & Figure S5). Since recruitment to IR–induced DNA damage sites is dependent on interaction with RAP80 and MERIT40 (Feng et al., 2009; Shao et al., 2009; Wang et al., 2009), the observed loss of localization of BRCC36 and Abraxas mutants was in agreement with the reduced association of both mutants in co-IP experiments (Figure 3A, C). Consistent with this notion, Abraxas mutants Phe20Ala and Glu237Tyr that retain interaction with RAP80 (Figure 3C) did not show a marked loss of IR–induced foci formation (Figure S5). Together, these results support the notion that the crystal structure of BRCC36–KIAA0157 reported here reflects a functionally relevant mode of interaction in cells.

### Catalytic and substrate binding infrastructure of BRCC36

Consistent with our observation of robust catalytic function in vitro, the active site residues in BRCC36 when complexed to KIAA0157 resided in an active-like conformation shared by AMSH-LP, CSN5 and RPN11 (Figure 2A and Figure S4A, B right panels). Productive features included the coordination of a high occupancy Zn^2+^ ion by the triad of His94, His96 and Asp107 side chains, the position of the transition state stabilizing Ser104 side chain, and water-coordinating catalytic Glu30 side chain. The active-like orientation of these key residues was supported by productive conformations of underlying secondary structure elements including strand β4 (denoted as the H-strand for positioning His94 and His96), helix α3 (denoted as the D-helix for positioning Asp107), the β4-α3 loop (denoted as the S-loop for positioning Ser104), and the α1- β2 loop (denoted E-loop for positioning Glu30).

With only one notable difference, the substrate recognition infrastructure of BRCC36 appeared generally similar to that of AMSH (Figure S4D-F), not unexpectedly since both display a strong preference for cleaving K63 linked ubiquitin chains. Indeed, the projected substrate contact site on BRCC36 is the most highly conserved surface across BRCC36 orthologues (Figure 2B). As a matter of convention, for a DUB engaging a diUb substrate of specific linkage type, the distal ubiquitin refers to the protomer that contributes its C-terminus, while the proximal ubiquitin refers to the protomer that contributes the Lys residue to the inter-ubiquitin isopeptide bond. AMSH-LP contains two essential insertions, Ins-1 and Ins-2 that contribute substantially to substrate contact (Sato et al., 2008). In AMSH-LP, Ins-1 contacts both the distal ubiquitin (buried SA = 1202 Å^2^) and, together with the short β4-α3 linker, the proximal ubiquitin centered on the lysine 63 linkage site (buried SA= 630 Å^2^) (Figure S4E). In BRCC36, a similarly configured Ins-1 (residues 53-83 rigid body shifted ~3.3 Å in the absence of substrate) and β4-α3 linker (residues 97-104), were well positioned to engage substrate in a K63-chain specific manner (Figure S4E). In AMSH-LP, Ins-2 mediates a smaller contact (buried SA = 387 Å^2^) with the proximal ubiquitin protomer remote from the K63 linkage site (Figure S4E). In BRCC36, the entire Ins-2 module was absent, with no possible compensatory contacts afforded by KIAA0157 (Figure S4D).

To confirm our inference of the extended substrate-binding surface on BRCC36, we mutated conserved residues on the projected distal ubiquitin (Met117) and proximal (Ile99) ubiquitin contact sites and measured their effects on catalytic function (Figure 2B and Figure S4F). Both Met117Ala and Ile99Arg mutants were greatly compromised for DUB activity approaching the complete loss of function resulting from an active site (Glu30Ala) mutation (Figure 2C). Together, these results suggest that despite lacking Ins-2, BRCC36 engages K63-linked diUb in a manner generally similar to AMSH-LP.

### Structural role of Zn and basis for KIAA0157 lack of catalytic function

KIAA0157 is predicted to lack catalytic function due to a deficiency of active site residues required to coordinate the catalytic Zn^2+^ ion (Maytal-Kivity et al., 2002). Consistent with this prediction, the projected active site region of CfKIAA0157 showed no evidence of a bound Zn^2+^ ion (Figure 1, Figure S6A). We attribute this to the substitution of the two essential Zn^2+^ coordinating residues (His94 and His96 in CfBRCC36) with Cys101 and Arg103 in CfKIAA0157. We note that the third triad position is maintained as Asp (position 115). Additional substitutions that would contribute to a lack of catalytic function include the substitution of the water-coordinating Glu side chain with Asn (residue 28) and a subtle substitution of the catalytic Ser residue with Thr (residue 112). Demonstrating that in addition to a catalytic role, that the Zn^2+^ ion in BRCC36 also plays a major scaffolding role, a BRCC36^QSQ^ mutant compromised for Zn^2+^ ion binding (harbouring His94Gln and His96Gln mutations shown previously to compromise DUB function in vitro and in cells (Patterson-Fortin et al., 2010; Sobhian et al., 2007), displayed extensive structural distortions to the active site region, including disorder of Ins-1, the S-loop and the D-helix (Figure S6B). In contrast, the corresponding elements were surprisingly ordered in KIAA0157. This may be attributable, in part, to compensating salt interactions in KIAA0157 involving the Arg103 and Asp115 projected catalytic triad positions. Suggesting that KIAA0157 has also dispensed with the ability to bind ubiquitin substrate, the position and sequence of Ins-1 in KIAA0157 deviated greatly from that in BRCC36 (Figure S6A).

### Mechanism by which KIAA0157 supports BRCC36 catalytic function

**Figure 4:**
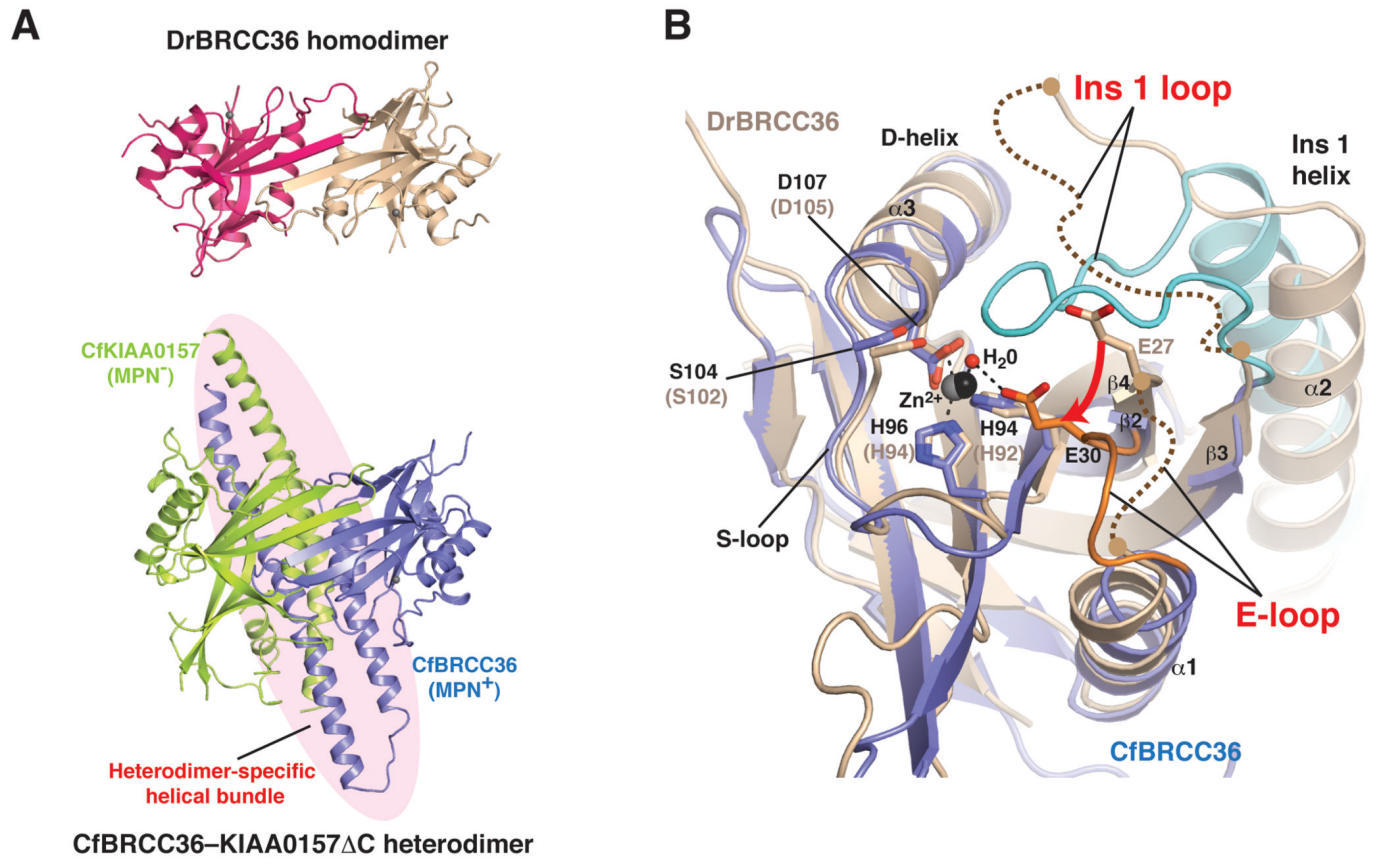
Comparison of the BRCC36 homodimer and BRCC36–KIAA0157 heterodimer **A)** Comparison of the DrBRCC36 homodimer (top panel) with the CfBRCC36–KIAA0157ΔC heterodimer (bottom panel) reveals similarities in MPN domain association but disorder of the helical bundle region. **B)** Zoom in of the active site region of CfBRCC36 (blue) superimposed on DrBRCC36 (wheat). The CfKIAA0157ΔC subunit complexed to CfBRCC36 has been omitted for clarity. Active site residues are shown as sticks and Zn^2+^ atoms and a catalytic water molecule are represented as spheres. Disordered regions are shown as curved dashed lines. See also Figures S2, S3 and S7.

To understand how KIAA0157 supports the catalytic function of BRCC36, we set out to solve an inactive-state structure of BRCC36 in isolation. Unlike CfBRCC36, which showed a propensity to aggregate at high protein concentrations, DrBRCC36 existed as a concentration dependent monomer-dimer in equilibrium (Figure S1E) and readily crystallized (see Table 1 for data collection and refinement statistics). Interestingly, DrBRCC36 adopted the analogous homodimer configuration involving the MPN domain (buried SA = 1693 Å^2^) displayed by the CfBRCC36-KIAA0157ΔC heterodimer (Figure 4A and Figure S7A, B for stereo view and combined sequence alignment), with the main exception that the CCHB helices α4 and α5 were completely disordered.

While the MPN^+^ domains of DrBRCC36 and CfBRCC36 were highly similar (RMSD of 2.0 Å over 140 Cα atoms), notable differences in the active site region of DrBRCC36 were apparent that might account for its lack of DUB activity in comparison to CfBRCC36 complexed to KIAA0157. In particular, the E-loop of the DrBRCC36 inactive structure was partially disordered and shifted with respect to the CfBRCC36 active structure, giving rise to a 4-5 Å displacement of the catalytic glutamate (Glu30 in CfBRCC36; Glu27 in DrBRCC36) (Figure 4B). Suggesting that this distortion was responsible in part for the loss of DUB activity (and not simply a trivial consequence of sequence divergence between ant and zebrafish proteins at 57% identity), the E-loop and the catalytic Glu residue reside in the same productive conformation in all deposited MPN^+^ X-ray structures that sample great variations of sequence identity (as low as 19%), species of origin (archaebacteria, yeast, zebrafish, and human) and substrate specificity (ubiquitin versus Nedd8 cleaving enzymes) (Figure S7C).

**Figure 5:**
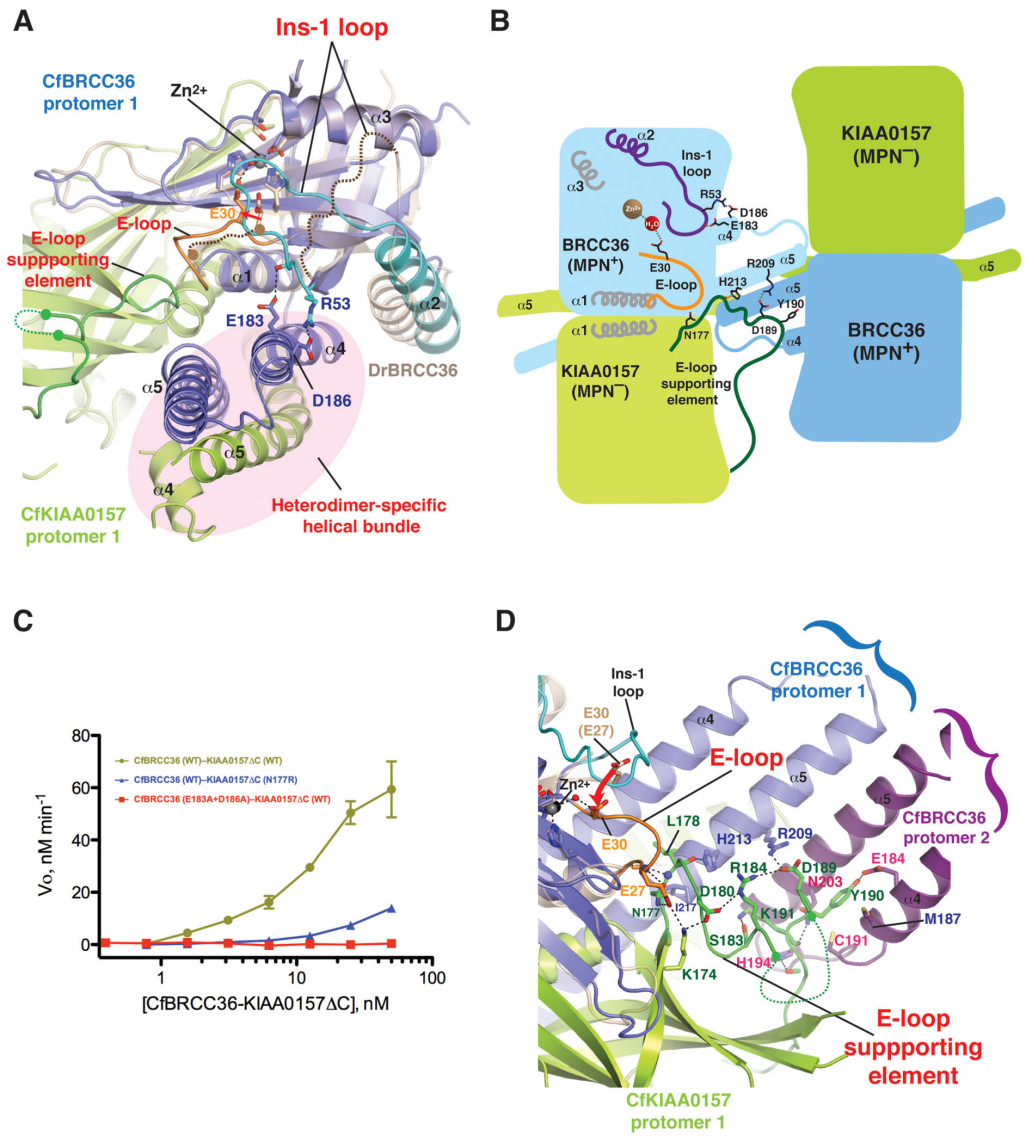
The active site conformation of BRCC36 is dependent on heterodimerization with KIAA0157 **A)** Zoom in comparison of the heterodimer interface of the CfBRCC36–KIAA0157ΔC complex (blue–green) superimposed on the homo dimerization interface of DrBRCC36 (wheat). The Ins-1 loop (cyan) of CfBRCC36 contacts helix α4 (blue) within the coiled coil helical bundle region (CCHB; highlighted by pink oval). Both regions in the DrBRCC36 homodimer are disordered. The E-loop of CfBRCC36 and E-loop supporting element of CfKIAA0157 are colored orange and green respectively. Disordered regions are shown as curved dashed lines. **B)** Schematic of inter-subunit interactions shown in panels A & D that support a productive conformation of the BRCC36 active site. The integrity of the helical bundle involving helices α4 and α5 of BRCC36 and KIAA0157 is dependent on BRCC36–KIAA0157 heterodimerization. Within one protomer of BRCC36, the Ins-1 loop is buttressed by contacts with helix α5. Across the BRCC36–KIAA0157 heterodimer, the E-loop of BRCC36 is buttressed by contacts with the “E-loop supporting element” of KIAA0157. The “E-loop supporting element” of KIAA0157 in turn is buttressed by contacts with helices α4 and α5 within the BRCC36–KIAA0157 heterodimer and also across the super dimer of BRCC36–KIAA0157 heterodimers. For clarity, detailed features are only drawn on one heterodimer. Not to scale. **C**) Cleavage activity of the WT CfBRCC36–KIAA0157ΔC complex and the indicated mutants towards an internally quenched K63-diUb fluorogenic substrate. Results ± SEM are the average of three independent experiments carried out in duplicate. Coomassie-stained SDS-PAGE and SEC-MALS analysis of the indicated complexes are shown in Figure S7D, E. **D)** Zoom in view of the heterodimer and super dimer interface of the CfBRCC36–KIAA0157ΔC complex (blue-green). The E-loop (orange) of CfBRCC36 contacts the E-loop supporting element (green) of CfKIAA0157. The E-loop supporting element of CfKIAA0157 in turn contacts the CCHB (specifically helix α5) of CfBRCC36 (denoted as protomer 1). In addition, the E-loop supporting element of CfKIAA0157 also makes contact to the CCHB of CfBRCC36 (purple-denoted protomer 2), of a second CfBRCC36-KIAA0157 heterodimer arranged in the super dimer configuration shown in Figs. 1B & 6A. Disordered regions in the DrBRCC36 MPN^+^ domain are shown as curved dashed lines. See also Figures S3, S7 and S8.

Additionally, a major portion of Ins-1 was disordered and the ordered portion (helix α2) was shifted relative to the corresponding position in CfBRCC36 (Figure 4B). Both of these structural distortions may be attributable to the loss of supporting interactions afforded by heterodimerization of CfBRCC36 with CfKIAA0157, as follows: Firstly, in the active heterodimer structure, a productive conformation of CfBRCC36 Ins-1 appears supported by direct contacts to CfBRCC36 helix α4, including between the invariant Arg53 (Ins-1) and Asp186 (α4) side chains and between the backbone carbonyl of Arg53 (Ins-1) and the invariant Glu183 (α4) side chain (Figure 5A, B and Figure S3A). The absence of the CCHB elements (specifically helix α4) in the inactive structure precluded formation of these interactions. Consistent with this inference, mutation of both Glu183 and Asp186 to alanine, selectively disabled CfBRCC36–KIAA0157 catalytic function (Figure 5C, Figure S7D, E). Secondly, a productive conformation of the E-loop, and in effect Glu30 of CfBRCC36, appears supported by direct contacts to the β7-α4 loop (residues 177-203) within the MPN^−^ domain of CfKIAA0157 (Figure 5B, D). We refer to this contact site on KIAA0157 as the E-loop supporting element. Specific interactions included between the invariant side chain of CfBRCC36 Glu27 and the backbone of Lys174 and Asn177 of CfKIAA0157, and between the backbone of CfBRCC36 Ser25 and CfKIAA0157 Leu178 (Figure 5B, D). Supporting the notion that these interactions have bearing on protein function, mutation of Asn177 to Arg in CfKIAA0157 selectively disabled CfBRCC36–KIAA0157 DUB activity (Figure 5C, Figure S7D, E). The position of the E-loop supporting element also appears to be aided by contacts between His213 and Ile217 in CfBRCC36 CCHB helix α5 and Leu178 of CfKIAA0157 (Figure 5D). Intriguingly, the E-loop supporting element of CfKIAA0157 also appears buttressed by interactions with CCHB helices α4 and α5 of CfBRCC36 from an opposing CfBRCC36–KIAA0157 heterodimer within the framework of the higher order super dimer (Figure 5B, D). These observations suggested a potential role for super dimerization in the attainment of the CfBRCC36 active state, a hypothesis we test below.

### Structural features of the BRCC36–KIAA0157ΔC super dimer

**Figure 6:**
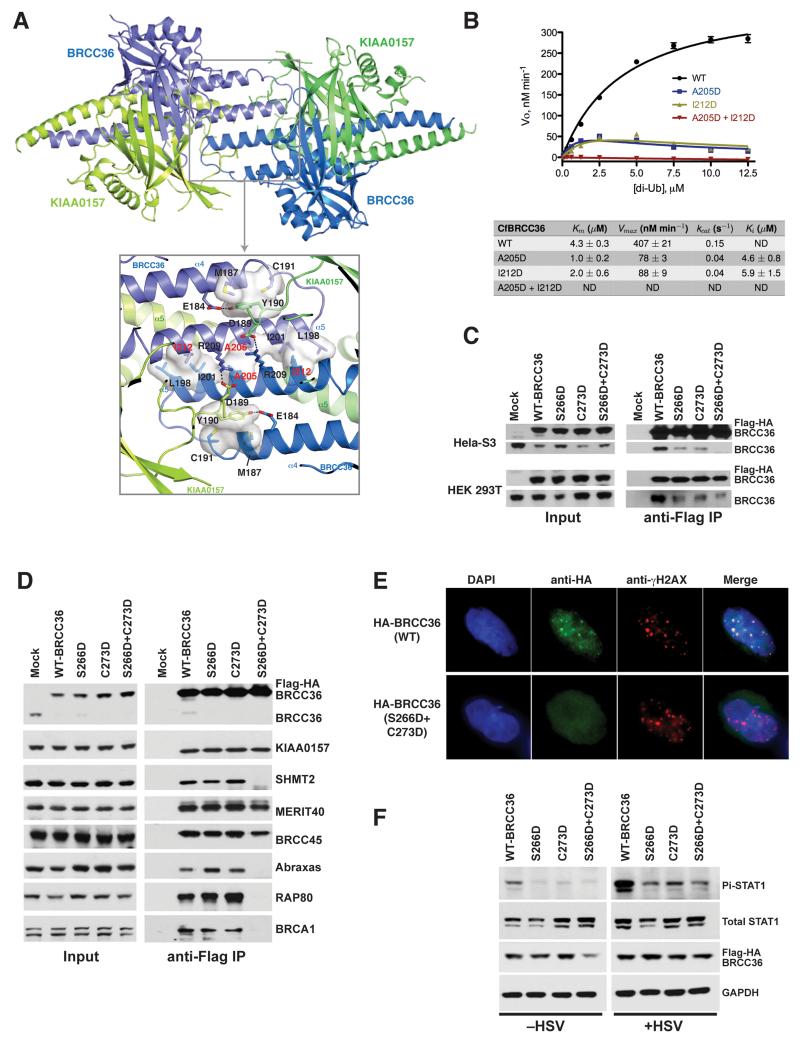
The super dimer configuration of BRCC36–KIAA0157 heterodimers is essential for function **A)** Ribbons view of a higher order dimer configuration (super dimer) of CfBRCC36–KIAA0157ΔC heterodimers. Zoom in panel shows a detailed view of interacting residues. Residues in red were mutated to test their contribution to super dimer formation. **B)** Michaelis-Menten kinetic analysis for cleavage of K63 diUb of wild type (44 nM) and the indicated super dimer formation defective mutants (35 nM) of CfBRCC36–KIAA0157ΔC. Results ± SEM are the average of three independent experiments carried out in duplicate. **C)** Flag- IP was performed in stably expressing HeLa S3 cells (top panels) or transiently transfected 293T cells (bottom panels) with either WT BRCC36 or super dimer defective mutants S266D, C273D and S266D + C273D. Immunoblot shows both ectopic and endogenous BRCC36 species from the co-IP for WT BRCC36, indicating multimerization. Reduced or absent association with endogenous BRCC36 was observed for the single and double mutated ectopic BRCC36. **D)** Flag-IP was performed on HeLa S3 cells stably expressing epitope tagged BRCC36 WT or mutants from (**C)**. Immunoblot was subsequently performed to assess interactions with the indicated members of BRISC and ARISC complexes. **E)** Co-localization of WT and S266D + C273D BRCC36 mutants with γH2AX in MEFs at 6 hours after 10 Gy IR. Foci that colocalize with γH2AX indicate recruitment to DNA double-strand breaks. Super dimer defective mutant S266D + C273D failed to exhibit IR induced foci formation. Quantification is shown in Figure S5. **F)** MEFs were stably transduced with Flag-HA- WT BRCC36 or the indicated super dimer formation defective mutants. Infection with HSV lacking ICP0 and immunoblot for Pi-STAT1 was performed as in Figure 3C to determine the impact on interferon signaling. See also Figures S1-S3 and S8.

Within the crystal environment, the functionally validated CfBRCC36–KIAA0157 heterodimer made four major contacts (>700 Å^2^) with surrounding heterodimers that we reasoned could be responsible for super dimerization in solution (Figure S8A). We collected small angle X-ray scattering data of the CfBRCC36–KIAA0157ΔC heterodimer at three different concentrations. The molecular weight of the complex calculated from the scattering curve was 126 kDa (Rambo and Tainer, 2013), consistent with the formation of a super dimer. *Ab initio* modeling and comparison of the calculated scattering curves for the heterodimer and the four possible super dimers with the experimental data using CRYSOL (Svergun et al., 1995) was most consistent with the two-fold symmetric super dimer configuration centered on CfBRCC36 helix α5 that buried 3093 Å^2^ of surface area (Figure S8B-D and Figure 6A). Contacts were predominantly hydrophobic in nature involving reciprocal patches composed by Leu198, Ile201, Ala205 and Ile212 side chains on opposing CfBRCC36 protomers (Figure 6A). Additional interactions were provided by loop β7-α4 of KIAA0157 (E-loop supporting element), most notably the invariant Tyr190 side chain, which inserted into a hydrophobic pocket between α4 and α5 and formed a hydrogen bond with the invariant Glu184 side chain of CfBRCC36 from the opposing heterodimer (Figure 6A). Finally, residues Arg209 (CfBRCC36) and Asp189 (CfKIAA0157) from the same heterodimer formed a salt bridge that tethered helix α5 of BRCC36 and the E-loop supporting element of CfKIAA0157.

### A super dimer of BRCC36–KIAA0157 is required for DUB activity in vitro and biological function in cells

BRCC36 DUB activity requires heterodimerization with KIAA0157. To determine the importance of the observed higher order assembly to this minimally active heterodimeric pair, we generated point mutations predicted to disrupt super dimerization without directly impacting on heterodimerization. As assessed by SEC-MALS, in contrast to the WT CfBRCC36–KIAA0157ΔC complex which eluted as a single peak with measured MW of 121.4 kDa corresponding to a super dimer (Figure S8E-G), the double CfBRCC36 mutant Ala205Asp + Ile212Asp eluted predominantly as a monomer of heterodimers (85% major peak; MW = 66.8 kDa) with only a minor fraction eluting as super dimers (15% minor peak; MW = 118.3 kDa). The single site mutants Ala205Asp and Ile212Asp of CfBRCC36 displayed intermediate behaviors with 70% and 36% of proteins, respectively, eluting as monomers of heterodimers (Figure S8E-G). These observations validate the mode of dimerization via BRCC36 helix α5 observed in the crystal structure (Figure 6A) as relevant for super dimerization in solution.

We next assayed WT and the super dimerization interface mutants for the ability to cleave a K63-linked diUb fluorigenic substrate in vitro (Figure 6B). In contrast to WT CfBRCC36–KIAA0157ΔC, which displayed robust enzyme activity, the double Ala205Asp + Ile212Asp BRCC36 mutant was completely devoid of DUB activity, and the single site mutants Ala205Asp and Ile212Asp mutants were ~10 fold less active than WT at near saturating substrate concentrations (>10 μM diUb; Figure 6B). In addition, the single site mutants displayed evidence of substrate inhibition, which when modeled, allowed the determination of full kinetic parameters (Figure 6B). These results showed that BRCC36 and KIAA0157 form a previously undescribed super dimer centered on BRCC36 helix α5 that is required for DUB activity in vitro, and that may influence substrate recognition properties.

To test whether super dimer formation occurred in the context of a cellular environment, Flag-HA-human BRCC36 and α5 single mutants Ser266Asp, Cys273Asp or the double mutant Ser266Asp + Cys273Asp (Table S2) were stably expressed in HeLa S3 cells (and transiently expressed in HEK 293T cells) and immunoprecipitated to assess interaction with endogenous BRCC36. Consistent with super dimer formation in cells, WT BRCC36 pulled down endogenous BRCC36, and this interaction was reduced for either single site mutant and almost eliminated for the double site mutant of BRCC36 (Figure 6C). Interestingly, requirements for super dimer formation were subtly different between the BRISC–SHMT2 and ARISC–RAP80 complexes (Figure 6D). The double BRCC36 mutant maintained all monomeric interactions in BRISC, but failed to show interferon induced interaction with SHMT2. Conversely, the double mutant failed to interact with Abraxas, BRCA1 and RAP80, and as predicted, did not form foci in response to IR (Figure 6E & Figure S5A). Moreover, consistent with the loss of DUB activity and interaction with SHMT2, expression of the Ser266Asp, Cys273Asp, or the double Ser266Asp + Cys273Asp mutants in MEFs showed reduced STAT1 phosphorylation in response to HSV infection (Figure 6F). These findings revealed that super dimer formation has a multifunctional purpose: it is required for enzymatic activity and for association with the targeting elements SHMT2 and RAP80. Abrogation of super dimer formation thus eliminates BRCC36 DUB activity in vitro and biological function in cells.

### Implications of super dimerization on substrate binding

**Figure 7:**
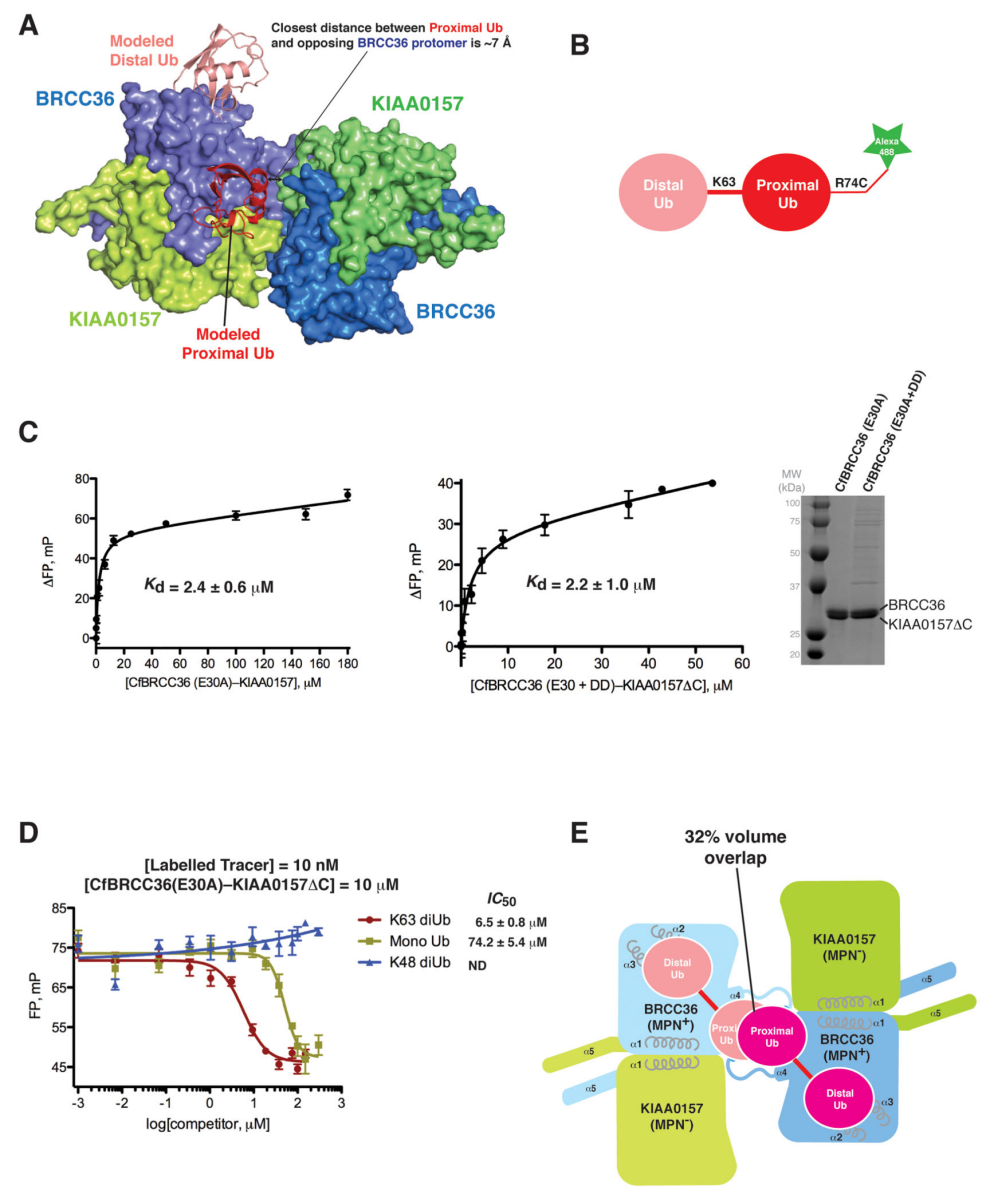
Binding of BRCC36–KIAA0157ΔC to K63-linked diUb is not dependent on super dimerization **A)** Modeling of K63-linked diUb to BRCC36 using the AMSH-LP–diUb co-structure (PDBID 2ZNV) as template. **B)** Schematic of K63-linked diUb covalently coupled to AlexaFluor-488. **C)** (left) Interaction of BRCC36 (E30A)–KIAA0157ΔC to the K63-linked diUb tracer (10 nM) as determined by fluorescence polarization (FP). *K*_d_ value ± SEM is the average of three independent experiments carried out in duplicate. (middle) Interaction of BRCC36 (E30A+A205D+I212D)–KIAA0157ΔC (super dimer breaker mutant) to the K63-linked diUb tracer (10 nM) as determined by fluorescence polarization (FP). *K*_d_ value ± SEM is the average of two independent experiments carried out in duplicate. (right) Coomassie-stained SDS-PAGE analysis of the indicated CfBRCC36–KIAA0157ΔC complexes. DD = CfBRCC36 A205D + I212D mutation. **D)** Displacement of AlexaFluor-488 labeled K63-linked diUb tracer by unlabeled K63-diUb, Mono-Ub and K48 diUb. Results ± SEM are the average of three independent experiments carried out in duplicate. **E)** Modeling of concurrent binding of diUb to opposing BRCC36 protomers within a BRCC36-KIAA0157 super dimer leads to a 32% volume overlap between proximal ubiquitin moieties.

The revelation of substrate inhibition-like behavior by the Ala205Asp and Ile212Asp single site mutants led us to investigate how a super dimer of BRCC36–KIAA0157 heterodimers might influence the engagement of K63-linked diUb substrate. Docking of K63 linked diUb substrate onto the BRCC36-KIAA0157 super dimer revealed a close approach of ~7 Å between the proximal Ub and the opposing BRCC36 protomer across the super dimer (Figure 7A). We reasoned that a subtle repositioning of the proximal ubiquitin moiety relative to the substrate bound AMSH-LP structure could afford unique and essential binding contacts for substrate engagement by BRCC36. To test this hypothesis, we compared the binding affinity of WT and a super dimer breaker mutant of BRCC36 to a fluorescently labeled K63 diUb substrate (Figure 7B) using a fluorescence polarization (FP) assay. To prevent cleavage of the fluorescent probe, we employed the E30A mutation within the catalytic site of both proteins. WT BRCC36(E30A)-KIAA0157ΔC complex bound substrate with a *K*_d_ of 2.4 +/− 0.6 μM (Figure 7C), in close agreement with the observed *K*_m_ of 4.2 μM (Figure 6B). Binding to the fluorescent probe was competed by unlabeled K63 linked diUb and to a lesser degree mono ubiquitin (*IC*_50_ of 6.4 vs 74 μM) but not K48 linked diUb (Figure 7D), consistent with the known specificity of the enzyme. The dimer breaker mutant complex BRCC36 (E30A+A205D+I212D)-KIAA0157ΔC showed no impairment of binding to K63 linked substrate (*K*_d_ of 2.2 +/− 1.0 μM; Figure 7C), ruling out essential contributions across the super dimer for substrate engagement. Together with the aforementioned conservation and mutational analyses (Figure 2B, C), these data support a model for substrate recognition by BRCC36 that is similar to AMSH-LP. Interestingly, engagement of diUb at both BRCC36 active sites would lead to a major steric clash (32% volume overlap) that could only be alleviated by a wholesale repositioning of the proximal ubiquitins (Figure 7E). We reason that this conflict might prohibit concurrent binding of substrate to both BRCC36 protomers within a super dimer.

## DISCUSSION

Our protein expression and co-purification studies on ARISC and BRISC in insect cells, native mass spectrometry analysis of HsBRISC, crystallographic and SAXS analyses on the CfBRCC36–KIAA0157 complex, mutational and (in vitro and cellular) functional studies on ARISC and BRISC, support the minimal architecture model for BRISC shown in Figure S2 (boxed). This model is applicable to ARISC with only the substitution of the paralogous subunit of KIAA0157 with Abraxas (40% identity, 65% similarity). Two BRCC36–KIAA0157 heterodimers assemble as a higher order super dimer at the center of the holoenzyme. Each KIAA0157 subunit recruits a BRCC45 subunit (as inferred by mass spectrometry Figure S2B**,** red oval), which in turn recruits a MERIT40 subunit.

The pseudo DUBs KIAA0157 and Abraxas are required for the catalytic function of BRCC36. Comparison of the BRCC36–KIAA0157 heterodimer structure with an inactive BRCC36 homodimer structure provides a model for understanding how this functional interplay is achieved. As shown in schematic form in Figure 5B**,** the binding of KIAA0157 to BRCC36 is required for stabilization of (1) the CCHB, (2) the E-loop of BRCC36 and optimal positioning of the catalytic glutamate side chain for catalysis, and (3) the Ins-1 loop of BRCC36 and its proper positioning for substrate binding. Stabilization of all three elements is supported through direct interactions between KIAA0157 and BRCC36 within a single heterodimer as well as through interactions across BRCC36–KIAA0157 heterodimers within a super dimer. Despite the modeled vicinity of the proximal ubiquitin moiety to the opposing BRCC36 protomer within the super dimer (Figure 7A), our data demonstrates that super-dimerization does not contribute positively to substrate recognition (Figure 7C).

In addition to supporting the catalytic function of BRCC36, KIAA0157 and Abraxas also serve scaffolding functions to recruit the BRCC45–MERIT40 heterodimer and the targeting subunits RAP80 or SHMT2. Surprisingly, super dimerization is not only required for the catalytic function of BRCC36 but it is also required for the binding of SHMT2 and RAP80/BRCA1 to BRISC and ARISC. It is also evident from modeling and mutational studies that super dimerization of BRISC/ARISC may impinge on enzyme function by restricting substrate binding to only one BRCC36 protomer in the super dimer at one time (Figure 7E). These inter-dependencies of structure and function provide intriguing potential for enzyme regulation and we speculate they may be responsible, in part, for the pronounced sensitivity to substrate inhibition displayed by the Ala205Asp and Ile212Asp single site dimer breaker mutants (Figure 6B).

The apparent obligate folding dependency of KIAA0157 and Abraxas on BRCC36 that we observed in insect cells hints that the heterodimer interaction is constitutive and may not require post-translational modifications. However, the super dimerization of BRISC (and by extension ARISC) may be regulated in principle, since mutations that drive a monomer state are well tolerated in our insect cell expression studies with no evidence of protein aggregation. If BRISC transitions between an inactive monomer and active dimer states, and only the dimer state can recognize the targeting elements SHMT2 and RAP80, this would provide a tight mechanism to couple enzyme activation with the recruitment to biologically relevant substrates. How might super dimerization be regulated? Others have shown that residues in the BRCC36 E-loop and in the KIAA0157 E-loop supporting element are phosphorylated in cells (Phosida database (Gnad et al., 2011)). Phosphorylation at these sites is well placed to affect catalytic function directly or indirectly by influencing super dimerization.

Since super dimerization involves contributions of both KIAA0157/Abraxas and BRCC36 subunits, it may be possible to differentially regulate ARISC and BRISC enzyme activity with small molecules by specifically targeting the MPN^−^ subunit. This would permit selective intervention on ARISC biological function in the DNA damage response or BRISC involvement in inflammatory cytokine signaling, even though the two holoenzymes share the same catalytic subunits. Additionally, although both complexes share a common architecture, the relative contribution and strength of the component interactions within ARISC and BRISC differ to some degree, as evidenced by our mutational studies in cells. Moreover, KIAA0157 is sufficient to impart DUB activity to BRCC36, while Abraxas and BRCC45 are both necessary for minimal DUB activity in ARISC (Cooper et al., 2010; Feng et al., 2010; Patterson-Fortin et al., 2010). These differences in function might also be exploited for selective intervention by small molecules.

Consistent with a clear separation of biological function between ARISC and BRISC in cells, mutations in BRCC36 and Abraxas are prevalent in cancer genomes whereas mutations in KIAA0157 are rare. A survey of the Catalogue of Somatic Mutations in Cancer (COSMIC) consortium, revealed that to date, 37 substituting mutations were mapped to BRCC36 (>15000 samples sequenced) and 35 to Abraxas (>19000 samples sequenced) while only 1 mutation was mapped to KIAA0157 (>19000 samples sequenced) (Table S3). The mutations in BRCC36 and Abraxas map to both MPN and CCHB domains (and additionally for Abraxas, the regulatory C-terminal tail, deleted in the KIAA0157 crystallization construct), and encode both single site amino acid substitutions and truncations, many of which would be predicted to cause loss of protein function. In contrast, only one mutation mapped to KIAA0157, and it resided outside the MPN and CCHB domain without a predictable effect on function. These observations demonstrate a strong selection for the loss of ARISC function in cancers and conversely, no specific selection against BRISC function.

In terms of a broader perspective on DUB regulation, it is fascinating that many multi-subunit DUBs contain two different MPN domain proteins. This feature is reminiscent of a subset of eukaryotic protein kinases, which employ dimerization to allosterically regulate their enzyme activity. Considerable diversity is displayed in how different protein kinase families dimerize, ranging from side-to-side, back-to-back, and head-to-tail configurations of kinase domains displayed by the RAF, eIF2α and EGFR families (Lavoie et al., 2014). This diversity reflects differences in the underlying modes of catalytic regulation. In the case of the RAF family and EGFR family kinases, specific members like KSR and HER2 respectively have dispensed with catalytic function altogether, while maintaining the ability to transactivate other family members through dimerization (Lavoie et al., 2014). These specific examples parallel the operation of MPN^+^–MPN^−^ paired DUBs. We see that BRCC36 (MPN^+^) heterodimerizes with the MPN^−^ pseudo DUBs KIAA0157 or Abraxas using a mode of interaction that is very different from that of the CSN5–CSN6 and RPN8–RPN11 heterodimers. This likely reflects the fact that the mechanism by which KIAA0157/Abraxas support BRCC36 will differ from how CSN6 and RPN8 support/regulate the function of CSN5 and RPN11. As approximately 10% of DUBs are predicted to be pseudo DUBs (Nijman et al., 2005), more examples of DUBs regulated by inactive pseudo DUBs are likely to emerge. Understanding the scope of DUB–pseudo DUB pairings may reveal new insights into how these important enzymes are regulated and how they achieve isopeptide hydrolysis.

## MATERIALS AND METHODS

### General methods and buffers

Restriction enzyme digests, DNA ligations, and other recombinant DNA procedures were performed using standard protocols. Mutagenesis was performed using the QuikChange site-directed mutagenesis method (Stratagene) with the KOD polymerase (Novagen). DNA constructs used for transfection were purified from *E. coli* DH5α using Qiagen Plasmid kits according to the manufacturer’s protocol. All DNA constructs were sequence verified. Lysis buffer used for purifying proteins from insect cells was composed of 50 mM Tris-HCl (pH 7.6), 300 mM NaCl, 20 mM imidazole, 5% (v/v) glycerol, 0.075% (v/v) 2-mercaptoethanol, 1 mM benzamidine, 1 mM PMSF. Lysis buffer was supplemented with SigmaFast protease inhibitors (EDTA-free) at 1 tablet/100 ml (Sigma). Wash buffer A was the same as lysis buffer without PMSF and protease inhibitors. High salt wash buffer B was the same as buffer A with 500 mM NaCl. All protein concentrations were calculated using the Beer-Lambert law by measuring the absorbance at 280 nm. Theoretical extinction coefficients were calculated using the ProtParam tool at the ExPASy proteomics server (http://www.expasy.org).

See Supplementary Information for a detailed description of the methods and materials used in this study.

## Supplementary Material

Supp_material

## Figures and Tables

**Table 1:** Summary of data collection and structure refinement. Values for high resolution shells are shown in brackets. B36 = BRCC36, KIAA = KIAA0157, QSQ = BRCC36 H94Q + H96Q mutant.

	CfB36-KIAΔC	CfB36-KIAΔC	CfB36(QSQ)-KIAΔC	DrBRCC36
**Substitution**	None	SeMet	None	None
**Space Group**	P2_1_2_1_2_1_	P2_1_2_1_2_1_	P2_1_2_1_2_1_	P 6_2_22
**Wavelength (Å)**	1.28240	0.97920	1.28270	0.97920
**Unit cell (Å)**				
***a***	49.9	50.1	89.4	108.8
***b***	116.6	116.3	124.2	108.8
***c***	226.1	231.5	132.5	137.4
***α* = *β***	90*°*	90*°*	90*°*	90*°*
*** γ ***	90*°*	90*°*	90*°*	120*°*
**Molecules/asu**				
**BRCC36**	2	2	2	1
**KIAA0157**	2	2	2	–
**Resolution (Å)**	2.55 (2.65-2.55)	2.54 (2.7-2.54)	2.75 (2.85-2.75)	3.2 (3.3-3.2)
**Observed reflections**	277108 (30610)	243872 (39265)	103925 (9417)	85408 (7589)
**Unique reflections**	80922 (8974)	84223 (13506)	37786 (3622)	8474 (816)
**Redundancy**	3.4 (3.4)	2.9 (2.9)	2.8 (2.6)	10.1 (9.3)
***I/σI***	7.2 (1.0)	14.3 (1.1)	7.3 (0.8)	12.7 (1.5)
**Completeness (%)**	97.3 (98.0)	98.1 (97.3)	95.8 (93.2)	99.9 (99.9)
***R_meas_***	0.123 (149.4)	0.059 (123.5)	0.158	0.201
***CC*_1/2_ (%)**	(43.4)	(52.4)	(50.0)	(87.4)
***R_work,_ R_free_***	0.211, 0.259	0.189, 0.240	0.231, 0.277	0.214, 0.241
**Average *B*-factor (Å^2^)**	76.5	93.7	72.9	82.3
**RMSD from ideal geometry**				
**bonds (Å)**	0.002	0.003	0.002	0.002
**angles (*°*)**	0.505	0.554	0.493	0.527
**Ramachandran plot statistics**				
**Residues in favored region**	97.0%	96.1%	98.2%	954.14%
**Residues in allowed region**	2.8%	3.8%	1.7%	4.9%
**Residues in outlier region**	0.2%	0.1%	0.1%	0.0%
**PDB ID**	–5CW3	5CW4–	5CW5–	5CW6–

## References

[R1] Ambroggio XI, Rees DC, Deshaies RJ (2003). JAMM: A Metalloprotease-Like Zinc Site in the Proteasome and Signalosome. PLoS Biol.

[R2] Cooper EM, Cutcliffe C, Kristiansen TZ (2009). K63-specific deubiquitination by two JAMM/MPN+ complexes: BRISC-associated Brcc36 and proteasomal Poh1 - Cooper - 2009 - The EMBO Journal - Wiley Online Library. The EMBO ….

[R3] Cooper EM, Boeke JD, Cohen RE (2010). Specificity of the BRISC deubiquitinating enzyme is not due to selective binding to Lys63-linked polyubiquitin. J Biol Chem.

[R4] Deshaies RJ (2014). Structural biology: Corralling a protein-degradation regulator. Nature.

[R5] Dong Y, Hakimi M-A, Chen X, Kumaraswamy E, Cooch NS, Godwin AK, Shiekhattar R (2003). Regulation of BRCC, a holoenzyme complex containing BRCA1 and BRCA2, by a signalosome-like subunit and its role in DNA repair. Mol Cell.

[R6] Eletr ZM, Wilkinson KD (2014). Regulation of proteolysis by human deubiquitinating enzymes. Biochim Biophys Acta.

[R7] Feng L, Huang J, Chen J (2009). MERIT40 facilitates BRCA1 localization and DNA damage repair. Genes Dev.

[R8] Feng L, Wang J, Chen J (2010). The Lys63-specific deubiquitinating enzyme BRCC36 is regulated by two scaffold proteins localizing in different subcellular compartments. J Biol Chem.

[R9] Fitzgerald DJ, Berger P, Schaffitzel C, Yamada K, Richmond TJ, Berger I (2006). Protein complex expression by using multigene baculoviral vectors. Nat Methods.

[R10] Gnad F, Gunawardena J, Mann M (2011). PHOSIDA 2011: the posttranslational modification database. Nucleic Acids Res.

[R11] Husnjak K, Dikic I (2012). Ubiquitin-binding proteins: decoders of ubiquitin-mediated cellular functions. Annu Rev Biochem.

[R12] Kim H, Chen J, Yu X (2007). Ubiquitin-binding protein RAP80 mediates BRCA1-dependent DNA damage response. Science.

[R13] Komander D, Rape M (2012). The ubiquitin code. Annu. Rev. Biochem.

[R14] Komander D, Clague MJ, Urbé S (2009). Breaking the chains: structure and function of the deubiquitinases. Nat Rev Mol Cell Biol.

[R15] Lavoie H, Li JJ, Thevakumaran N, Therrien M, Sicheri F (2014). Dimerization-induced allostery in protein kinase regulation. Trends in Biochemical Sciences.

[R16] Lingaraju GM, Bunker RD, Cavadini S, Hess D, Hassiepen U, Renatus M, Fischer ES, Thomä NH (2014). Crystal structure of the human COP9 signalosome. Nature.

[R17] Maytal-Kivity V, Reis N, Hofmann K, Glickman MH (2002). MPN+, a putative catalytic motif found in a subset of MPN domain proteins from eukaryotes and prokaryotes, is critical for Rpn11 function. BMC Biochem.

[R18] Nijman SMB, Luna-Vargas MPA, Velds A, Brummelkamp TR, Dirac AMG, Sixma TK, Bernards R (2005). A genomic and functional inventory of deubiquitinating enzymes. Cell.

[R19] Pathare GR, Nagy I, Sledz P, Anderson DJ, Zhou HJ, Pardon E, Steyaert J, Forster F, Bracher A, Baumeister W (2014). Crystal structure of the proteasomal deubiquitylation module Rpn8-Rpn11. Proceedings of the National Academy of Sciences.

[R20] Patterson-Fortin J, Shao G, Bretscher H, Messick TE, Greenberg RA (2010). Differential Regulation of JAMM Domain Deubiquitinating Enzyme Activity within the RAP80 Complex. J Biol Chem.

[R21] Rambo RP, Tainer JA (2013). Accurate assessment of mass, models and resolution by small-angle scattering. Nature.

[R22] Sato Y, Yoshikawa A, Mimura H, Yamashita M, Yamagata A, Fukai S (2009). Structural basis for specific recognition of Lys 63-linked polyubiquitin chains by tandem UIMs of RAP80. Embo J.

[R23] Sato Y, Yoshikawa A, Yamagata A, Mimura H, Yamashita M, Ookata K, Nureki O, Iwai K, Komada M, Fukai S (2008). Structural basis for specific cleavage of Lys 63-linked polyubiquitin chains. Nature.

[R24] Shao G, Patterson-Fortin J, Messick TE, Feng D, Shanbhag N, Wang Y, Greenberg RA (2009). MERIT40 controls BRCA1-Rap80 complex integrity and recruitment to DNA double-strand breaks. Genes Dev.

[R25] Sims JJ, Cohen RE (2009). Linkage-specific avidity defines the lysine 63-linked polyubiquitin-binding preference of rap80. Mol Cell.

[R26] Sobhian B, Shao G, Lilli DR, Culhane AC, Moreau LA, Xia B, Livingston DM, Greenberg RA (2007). RAP80 targets BRCA1 to specific ubiquitin structures at DNA damage sites. Science.

[R27] Svergun D, Barberato C, Koch MHJ (1995). CRYSOL – a Program to Evaluate X-ray Solution Scattering of Biological Macromolecules from Atomic Coordinates. J Appl Crystallogr.

[R28] Tran HJTT, Allen MD, Löwe J, Bycroft M (2003). Structure of the Jab1/MPN Domain and Its Implications for Proteasome Function †. Biochemistry.

[R29] Wang B, Hurov K, Hofmann K, Elledge SJ (2009). NBA1, a new player in the Brca1 A complex, is required for DNA damage resistance and checkpoint control. Genes Dev.

[R30] Wang B, Matsuoka S, Ballif BA, Zhang D, Smogorzewska A, Gygi SP, Elledge SJ (2007). Abraxas and RAP80 form a BRCA1 protein complex required for the DNA damage response. Science.

[R31] Worden EJ, Padovani C, Martin A (2014). Structure of the Rpn11–Rpn8 dimer reveals mechanisms of substrate deubiquitination during proteasomal degradation. Nat Struct Mol Biol.

[R32] Zheng H, Gupta V, Patterson-Fortin J, Bhattacharya S, Katlinski K, Wu J, Varghese B, Carbone CJ, Aressy B, Fuchs SY (2013). A BRISC-SHMT Complex Deubiquitinates IFNAR1 and Regulates Interferon Responses. CellReports.

