# Supplementary material for: Higher -Order Assembly of BRCC36–KIAA0157 Is Required for DUB Activity and Biological Function
Source: Mol Cell. Author manuscript; Available in PMC 2015 Sep 23. (PMC4579573; doi:10.1016/j.molcel.2015.07.028)
Supplement: Supp_material [file NIHMS64874-supplement-Supp_material.pdf]

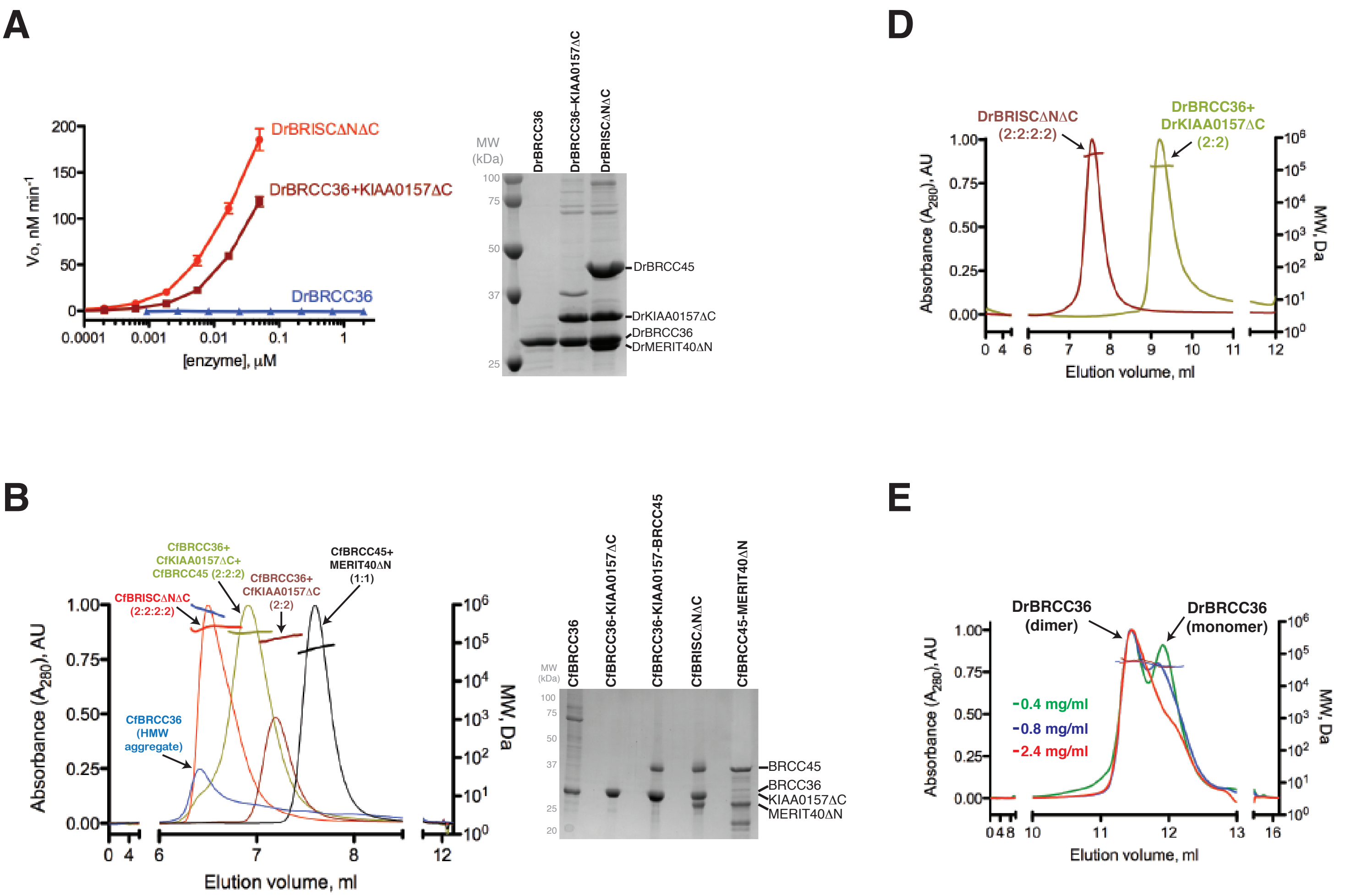

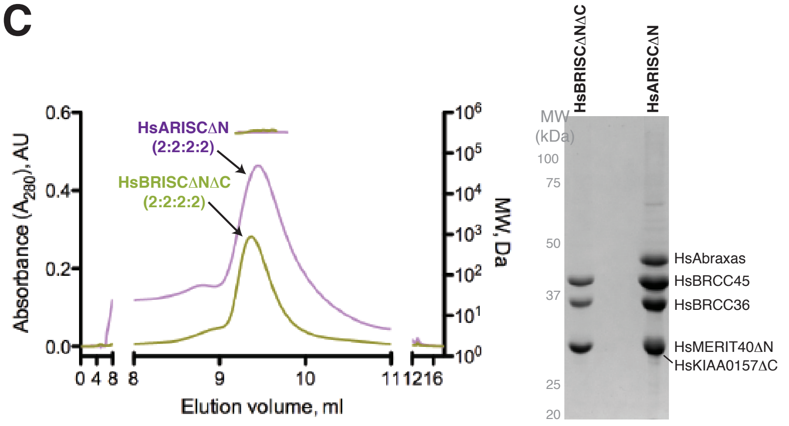

F

Protein	Measured MW (kDa)	Calculated MW (kDa)	Calculated MW for dimer (kDa)
CfBRCC36*	Aggregate	28.7	57.4
CfBRCC36-KIAA0157ΔC	131.5	61.2	122.4
CfBRCC36-KIAA0157ΔC-BRCC45	187.8	105.4	210.8
CfBRISCΔNΔC	255.8	132.5	265.0
CfBRCC45-MERIT40ΔN	90.7	71.3	142.6
DrBRCC36 (Peak 1)	56.7	29.6	59.2
DrBRCC36 (Peak 2)	42.7	29.6	59.2
DrBRCC36-KIAA0157ΔC	130.2	60.5	120.9
DrBRISCΔNΔC	269.8	133.1	266.2
HsBRISCΔNΔC	346.1	140.1	280.2
HsARISCΔNΔC	330.0	139.2	278.4

**CfBRCC36 elutes as a high molecular weight (HMW) aggregate*

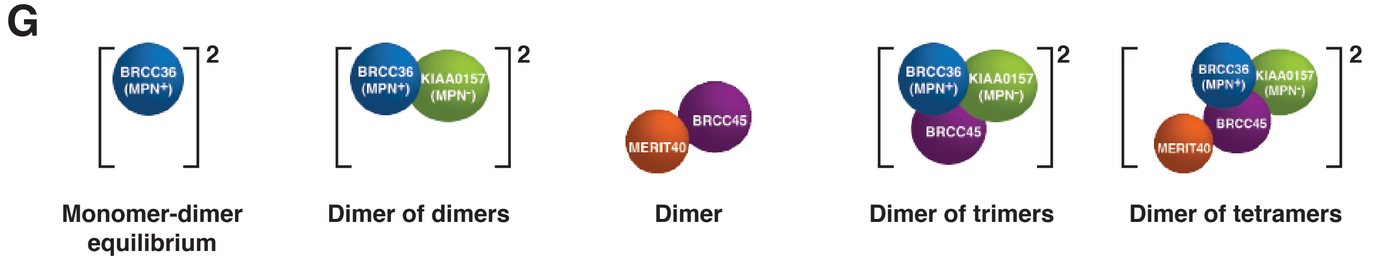

Figure S1

Related to Figure 1

A

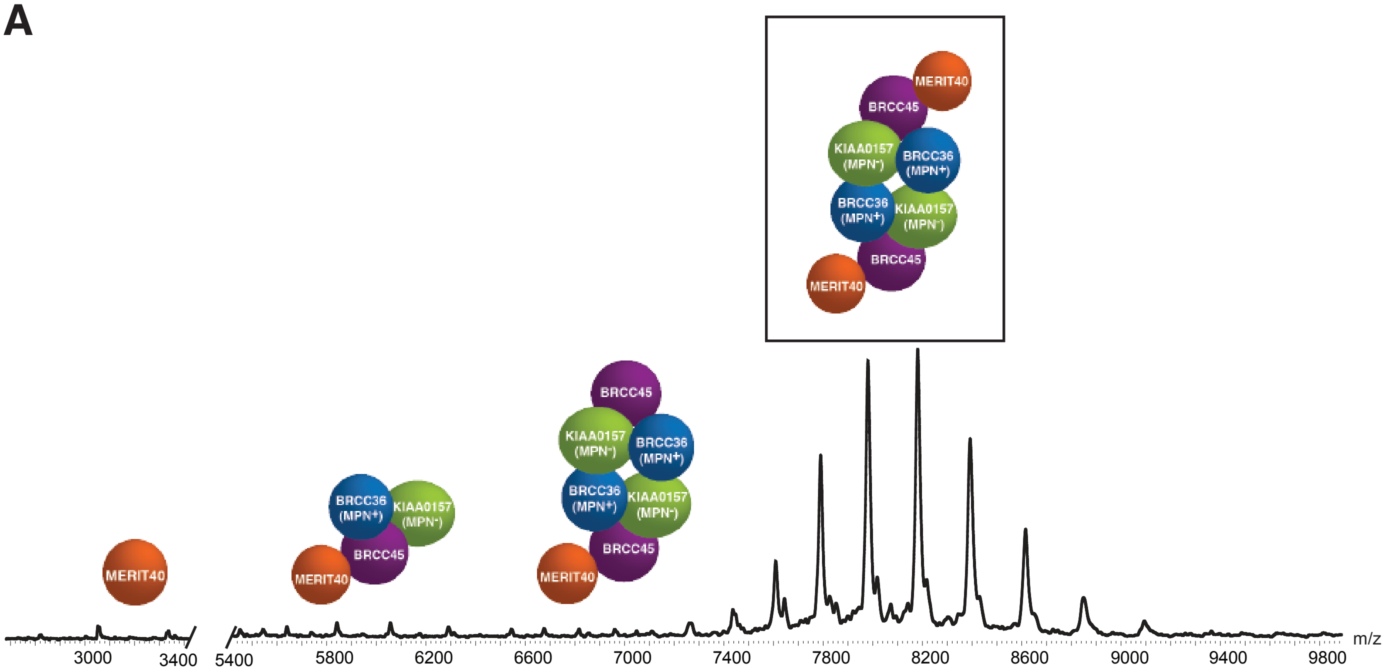

B

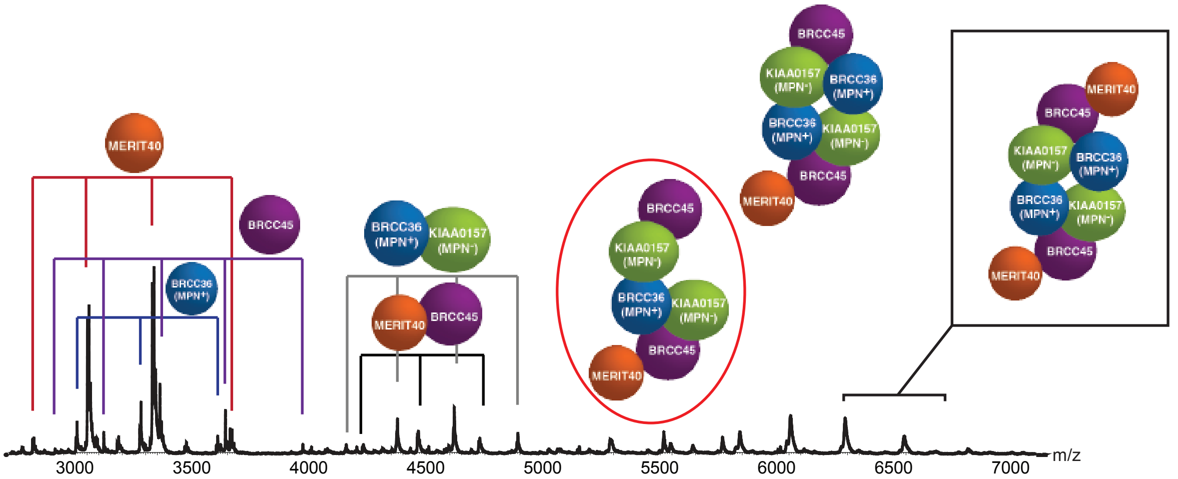

C

Stoichiometry	Subunit	Theoretical mass (Da)	Measured single subunit (Da)	Calculated complexes (Da)	Measured mass complexes (Da)
A	BRCC45	43680	43687		
B	BRCC36	36100	36079		
C	MERIT40	36560	36688		
D	KIAA0157	46904	47055		
AC		80240		80376	80375
BD		83004		83140	83134
ABD		126684		126837	126821
ABCD		163244		163493	163509
B_2D_2		166008		166258	166268
A_2B_2D		209688		209979	209955
A_2BC_2D		246248		246669	246643
$A_2B_2C_2D$		289928		290330	290331
$A_2B_2C_2D_2$		326488		326988	327018

D

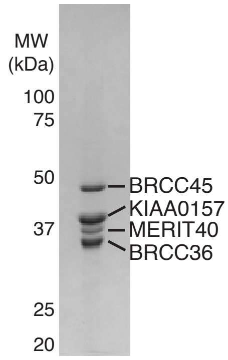

Figure S2

Related to Figure 1, Figure 4 and Figure 6

Continued on next page

Continued from previous page

E

CfBRCC36 **DrBRCC36**

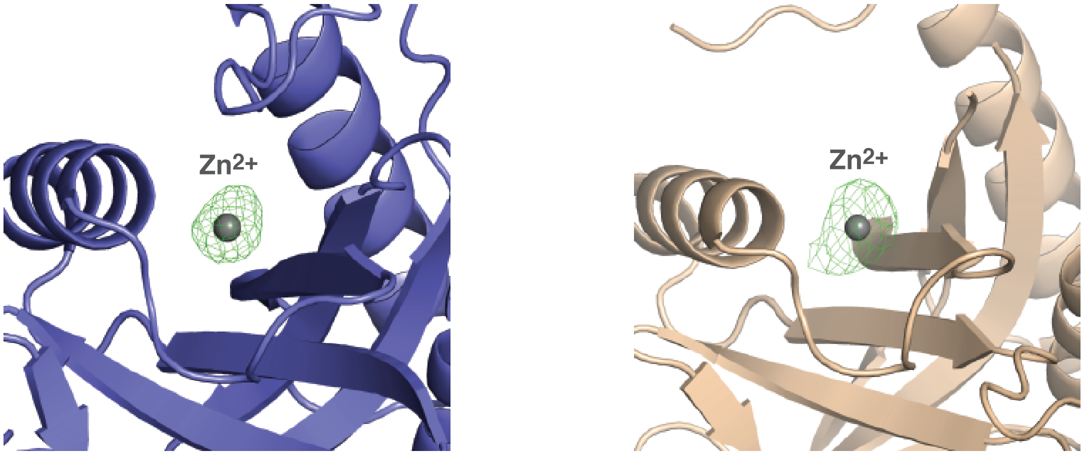

Unbiased F_o-F_c electron density maps at 2.5σ

F

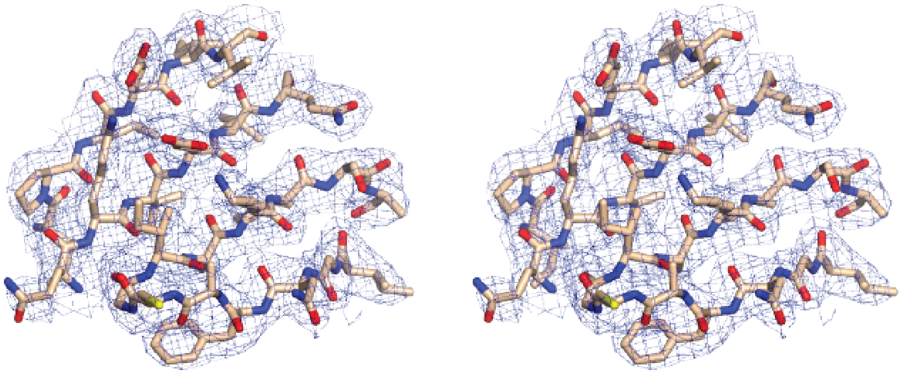

CfBRCC36

Final 2F_o-F_c electron density maps at 1.0σ

G

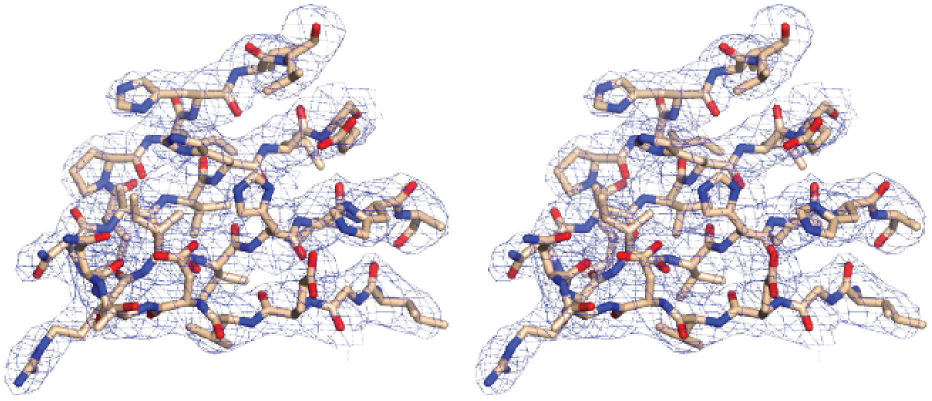

DrBRCC36

Final 2F_o-F_c electron density maps at 1.0σ

Figure S2

Related to Figure 1, Figure 4 and Figure 6

A

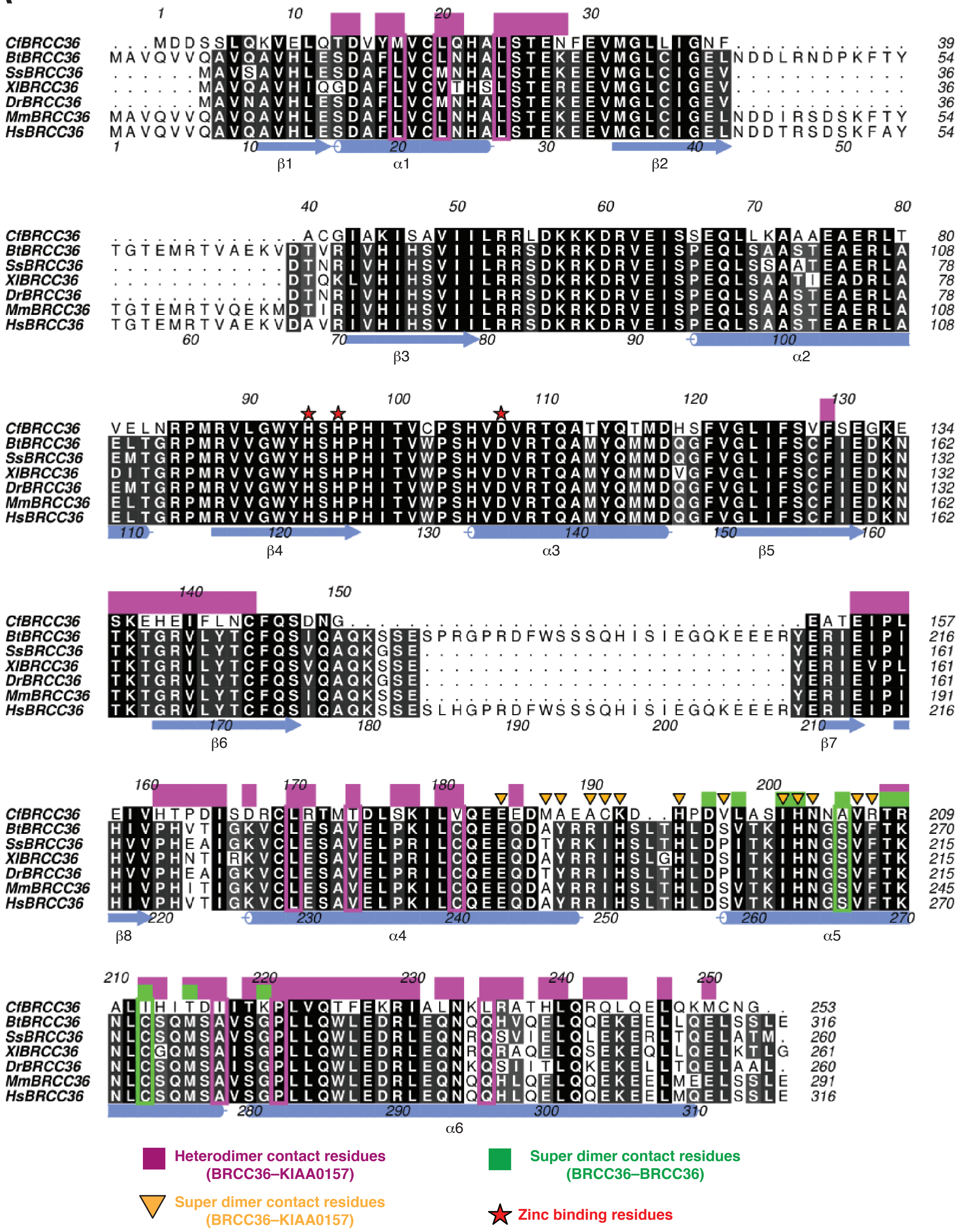

Figure S3

Related to Figure 1, Figure 2, Figure 3, Figure 4, Figure 5 and Figure 6

Continued on next page

Continued from previous page

B

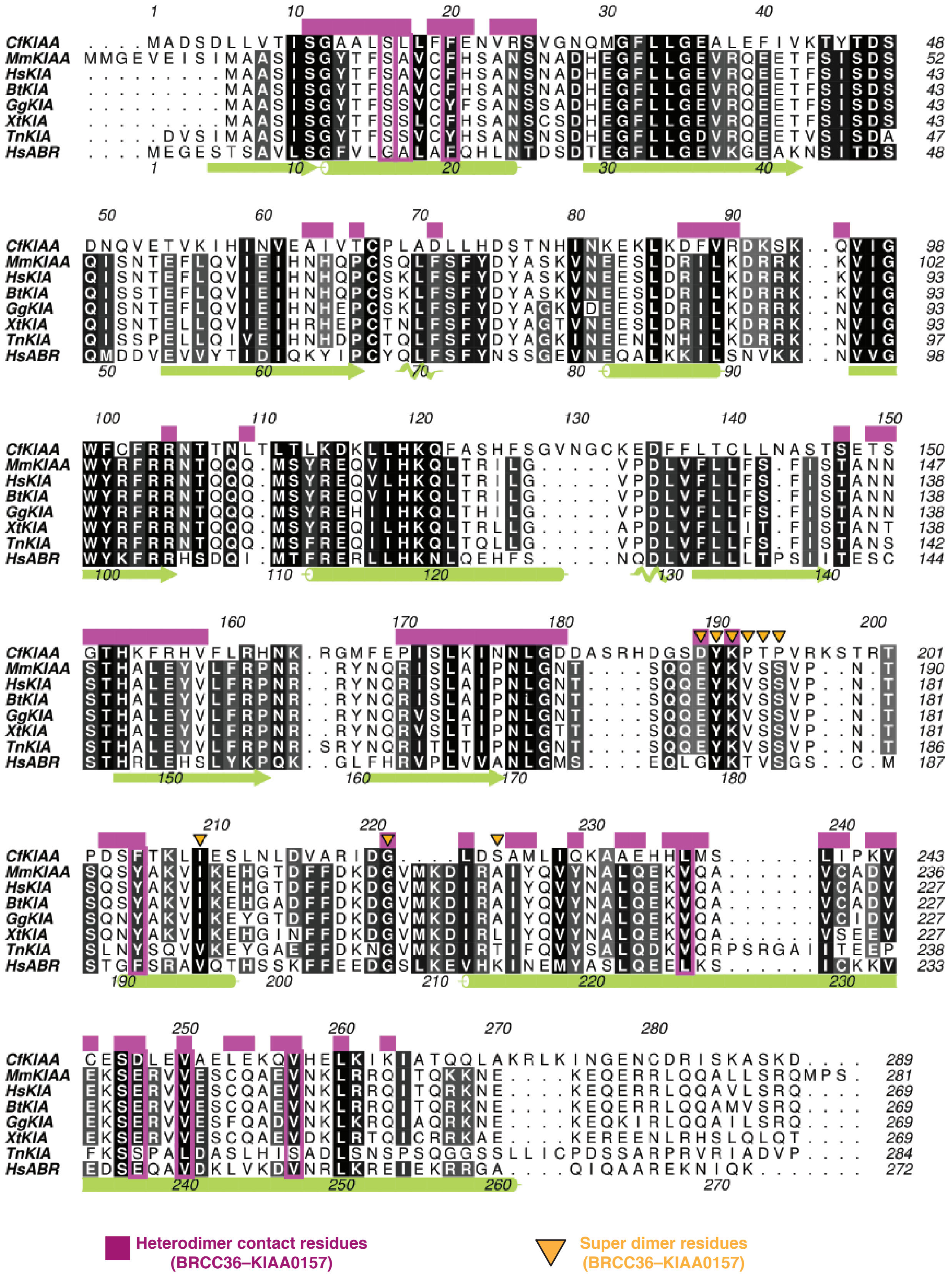

Figure S3

Related to Figure 1, Figure 2, Figure 3, Figure 4, Figure 5 and Figure 6

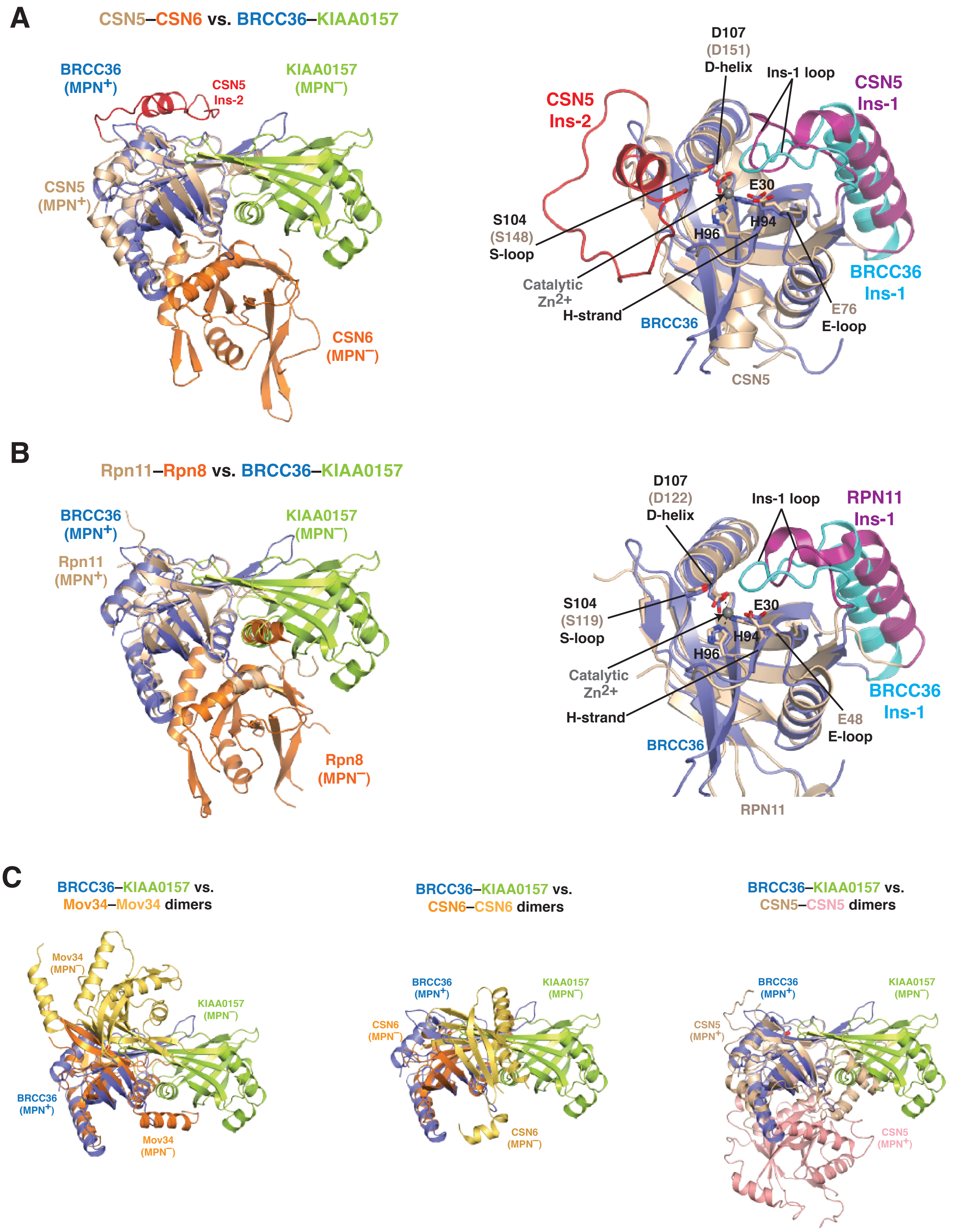

Figure S4

Related to Figure 1 and Figure 2

Continued on next page

Continued from previous page

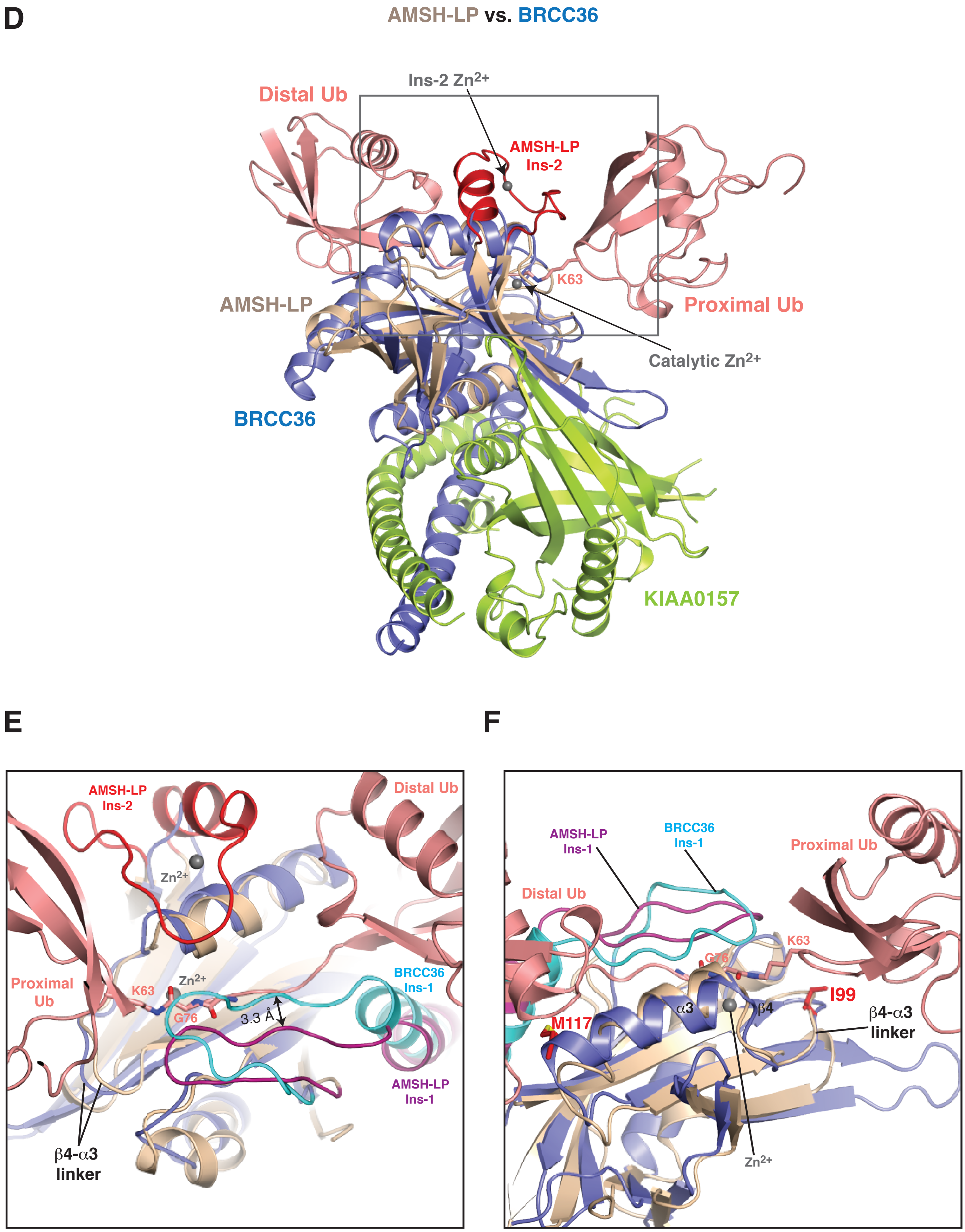

Figure S4
Related to Figure 1 and Figure 2

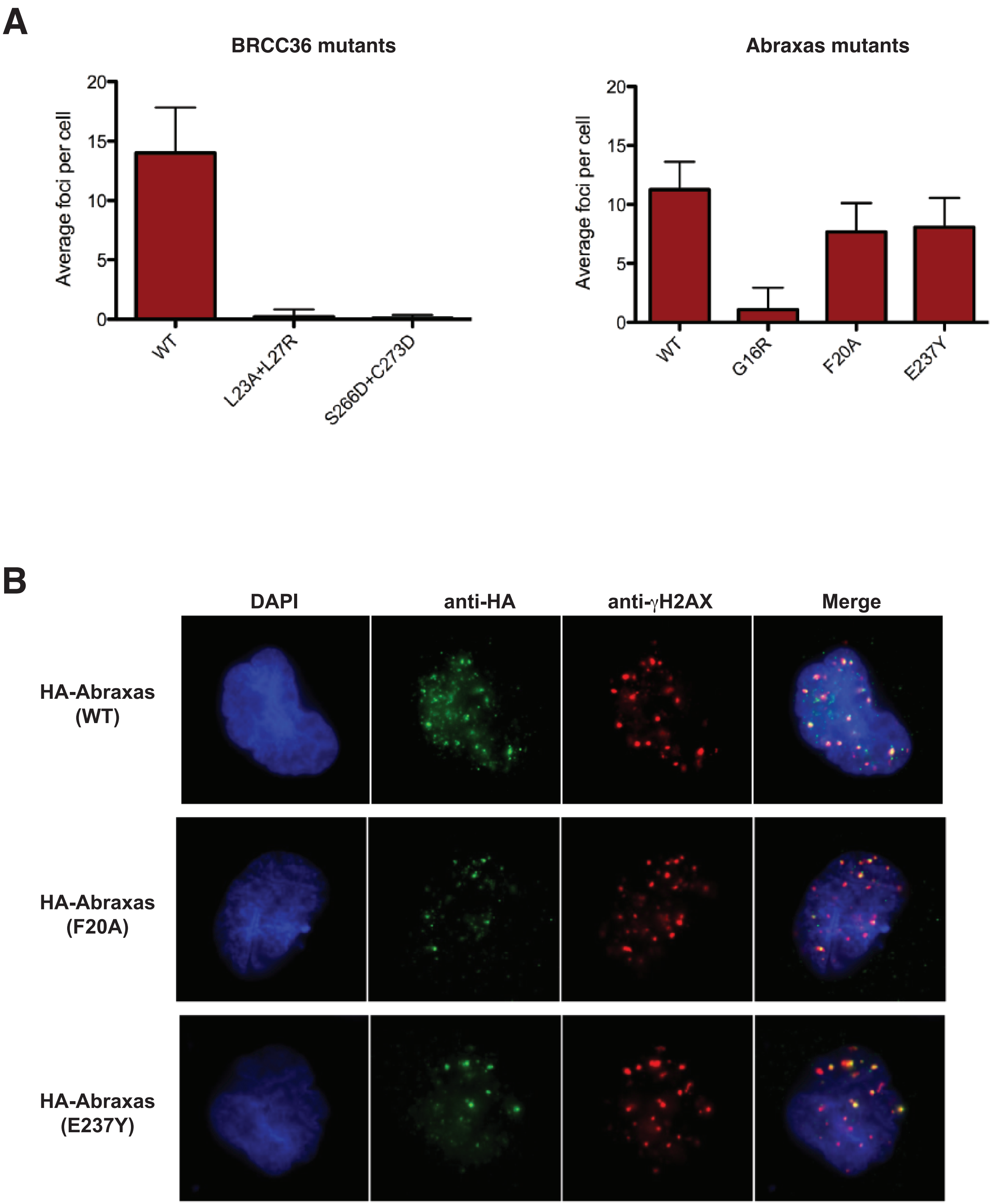

Figure S5

Related to Figure 3 and Figure 6

A

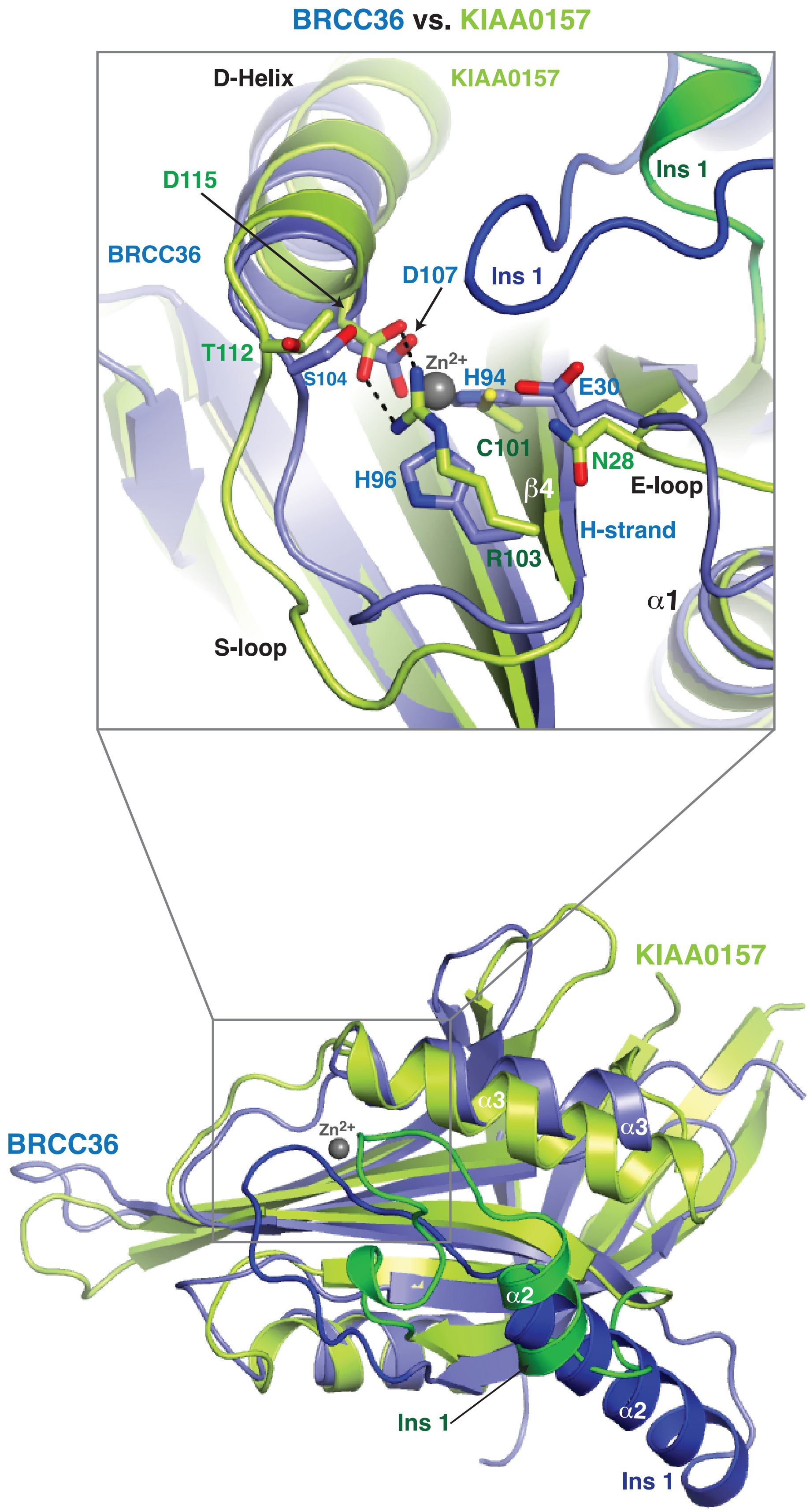

Figure S6
Related to Figure 2

Continued on next page

Continued from previous page

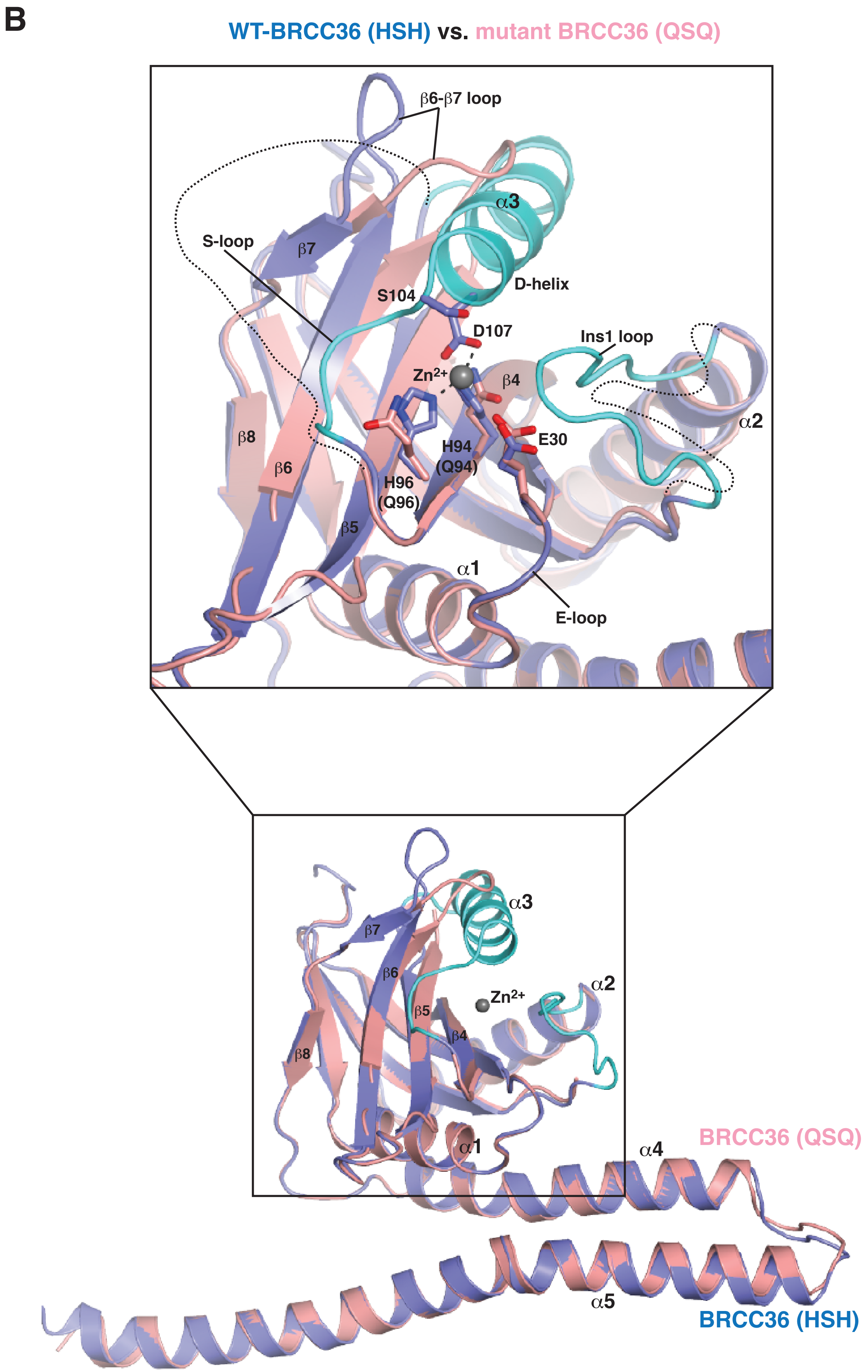

Figure S6
Related to Figure 2

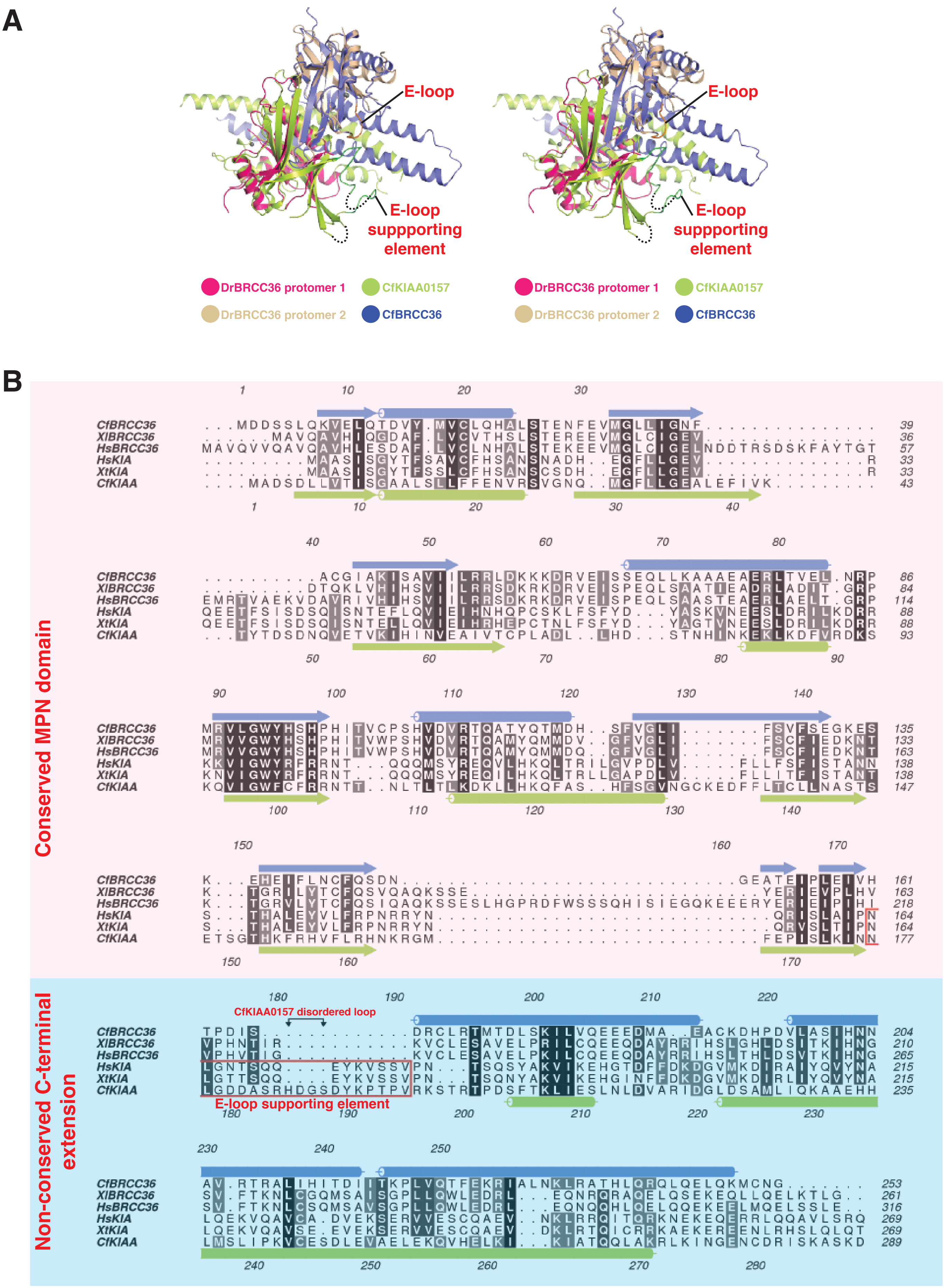

Figure S7

Related to Figure 4 and 5

Continued on next page

Continued from previous page

C

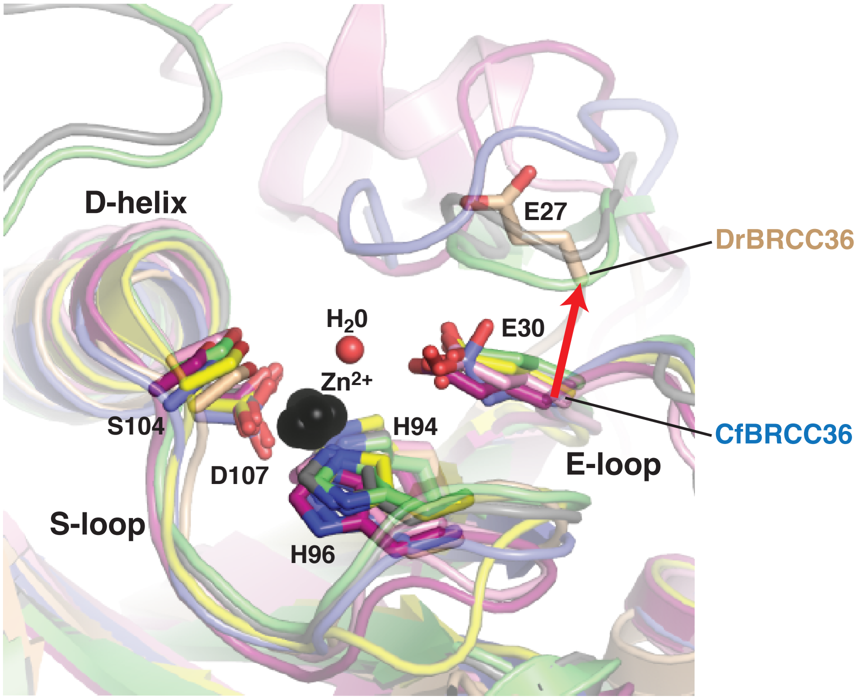

Species name	Protein name	% sequence identity to Cf (ant) BRCC36	PDB ID
Zebrafish	DrBRCC36	57. 0	–
Human	HsAMSH-LP	23.7	2ZNV
Human	HsCSN5	25.4	4F70
Archaebacteria	AfJAMM	21.6	1R5X
Yeast	ScSST2	18.8	4K1R
Yeast	ScRPN11	19.7	4O8Y

D

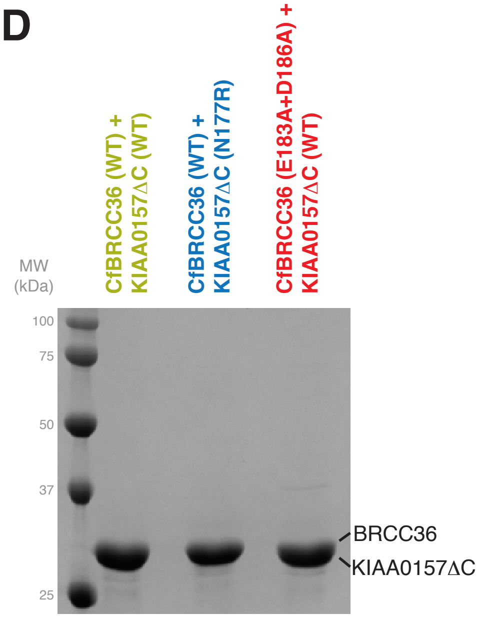

E

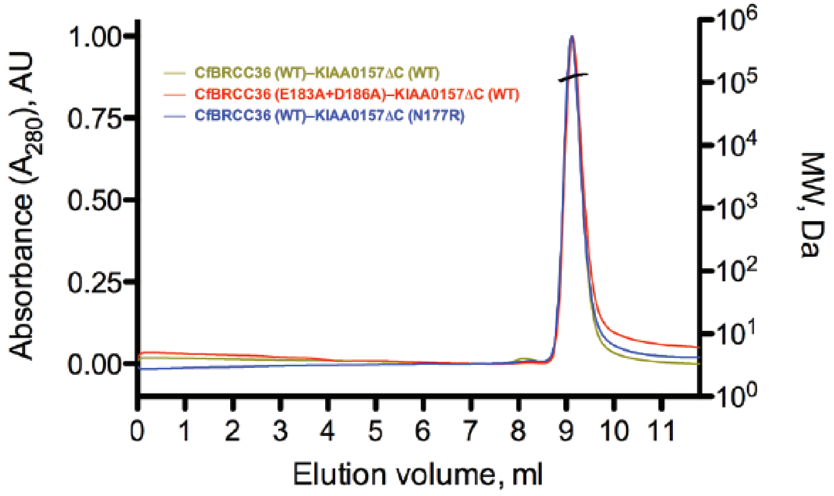

Figure S7

Related to Figure 4 and Figure 5

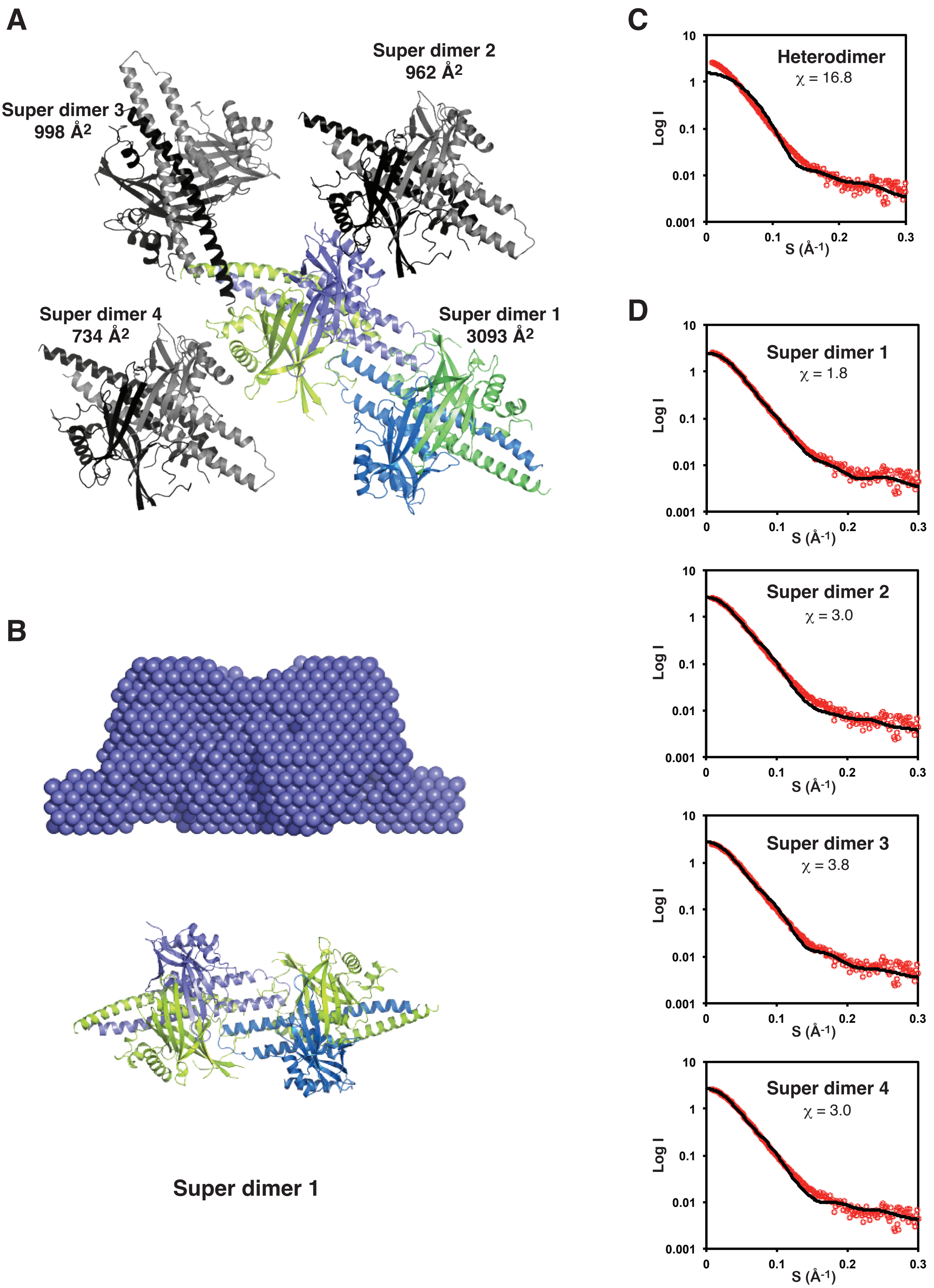

Figure S8
Related to Figure 6

Continued on next page

Continued from previous page

E

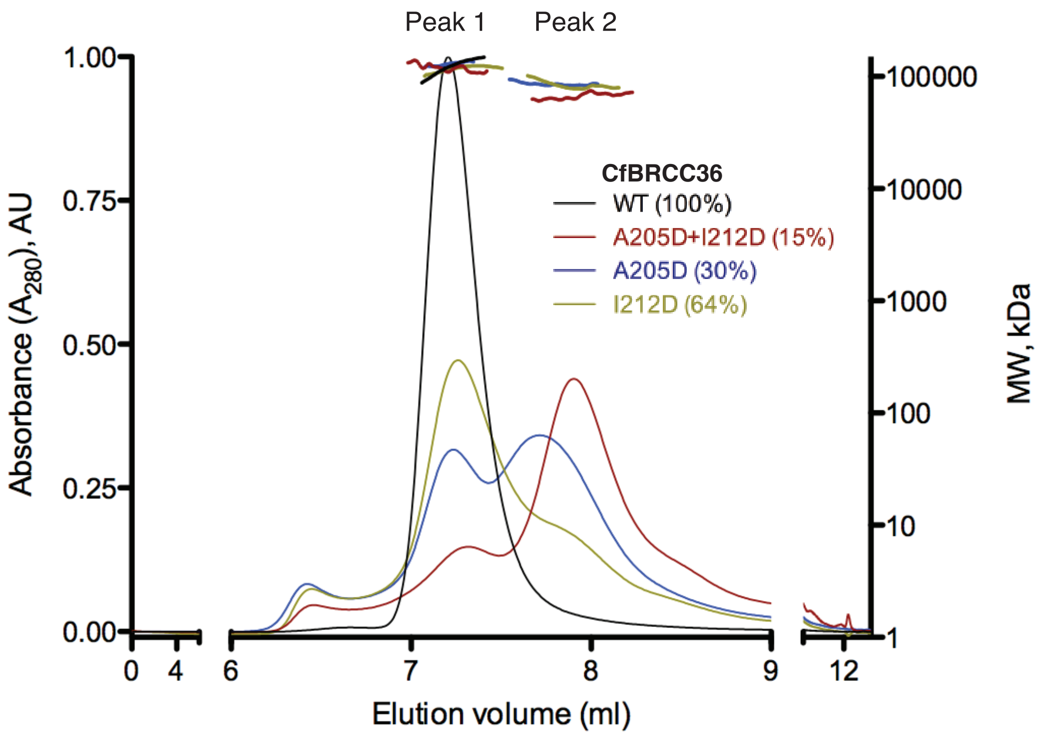

F

CfBRCC36	Peak1 Measured MW (kDa)	Peak2 Measured MW (kDa)	Calculated MW for 1:1 complex (kDa)
**WT**	121.4	ND	61.3
**A204D+I212D**	118.3	66.8	61.3
**A204D**	129.7	85.7	61.3
**I212D**	117.4	85.1	61.3

G

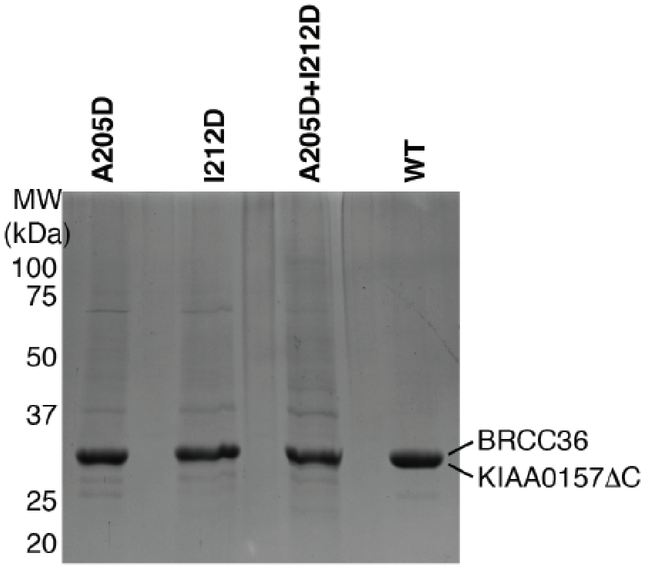

Figure S8

Related to Figure 6

SUPPLEMENTAL FIGURE LEGENDS

Figure S1. Analysis of BRCC36-containing complexes and subcomplexes

A) Cleavage activity of *D. rerio* BRCC36 and BRCC36-containing complexes towards an internally quenched K63-diUb fluorogenic substrate (left). Coomassie-stained SDS-PAGE analysis of the indicated complexes (right).

B) Size exclusion chromatography followed by multi angle light scattering (SEC-MALS) analysis (left) and Coomassie-stained SDS-PAGE analysis (right) of the indicated *C. floridanus* protein complexes.

C) SEC-MALS analysis (left) and Coomassie-stained SDS-PAGE analysis (right) of the indicated *H. sapiens* BRISC and ARISC protein complexes.

D & E) SEC-MALS analysis of the indicated DrBRCC36-containing complexes **(D)** and DrBRCC36 run at the indicated concentrations **(E)**.

F) Summary table of measured and theoretical calculated molecular weights for each protein and protein complex shown in panels **B, C, D** and **E**.

G) Cartoon model of predicted sizes and stoichiometry for the indicated complexes.

Figure S2. Native mass spectrometry measurements and crystallographic analyses of BRCC36-containing complexes and subcomplexes

A) Mass spectrum under gentle ionizing conditions. The primary distribution between 7400 and 9000 m/z fits with the mass of a complex with two copies of each BRCC45, BRCC36, MERIT40 and KIAA0157 subunits (2:2:2:2 stoichiometry). A cartoon representation of the complexes and binding topology consistent with the measured mass and with the assembly constraint of protein expression studies is shown above the spectrum.

B) Mass spectrum under conditions of in source fragmentation. Cartoons shown as in (**A**).

C) Summary table of measured and theoretical masses of individual proteins and their complexes.

D) Coomassie-stained gel of HsBRISC used for mass spectrometry analysis.

E) Unbiased F_o-F_c electron density maps contoured at 2.5σ showing Zn^{2+} bound to the active site of CfBRCC36 (left panel) and DrBRCC36 (right panel).

F) Stereo view of final 2F_o-F_c electron density maps for CfBRCC36.

G) Stereo view of final 2F_o-F_c electron density maps for DrBRCC36.

Figure S3. Sequence conservation of BRCC36, KIAA0157 and Abraxas

A) Multiple sequence alignment (black = conserved, white = not conserved) of BRCC36 from the indicated species. Alignments were performed with MUSCLE and edited and displayed using ALINE. Contact residues involved in the BRCC36–KIAA0157 interactions are shown above the alignment in purple. KIAA0157-interacting residues mutated in this study are boxed. The secondary structure determined by DSSP is shown in blue below the alignment. The ant and human BRCC36 sequences are numbered at the top and bottom of the alignment respectively. *Cf, C. floridanus; Bt, B. Taurus; Ss, S. scrofa; Xl, X. laevis; Dr, D. rerio; Hs, H. sapiens.*

B) Multiple sequence alignment (black = conserved, white = not conserved) of KIAA0157 (KIAA) and Abraxas (ABR) from the indicated species. Alignments were performed with MUSCLE and edited and displayed using ALINE. Contact residues involved in the BRCC36–KIAA0157 interactions are shown above the alignment in purple. BRCC36-interacting residues mutated in this study are boxed. The secondary structure determined by DSSP is shown in blue below the alignment. The ant KIAA0157 and human Abraxas sequences are numbered at the top and bottom of the alignment respectively. *Cf, C. floridanus; Mm, M. Musculus; Hs, H. sapiens; Bt, B. Taurus; Gg, G. gallus, Xt, X. tropicalis; Tn, T. nigroviridis.*

Figure S4. Comparison of the BRCC36–KIAA0157 complex with other higher order MPN domain structures

A) Left, comparison of the CfBRCC36–KIAA0157 and CSN5–CSN6 complexes (PDBID 4D10). Right, zoom in view of the superimposed MPN^+ active site regions.

B) Left, comparison of the CfBRCC36–KIAA0157 and Rpn11–Rpn8 complexes (PDBID 4O8X). Right, zoom in view of the superimposed MPN^+ active site regions.

C) Ribbons comparison of the CfBRCC36–KIAA0157ΔC heterodimer with (left) Mov34–Mov34 homodimer (PDBID 2O95), (middle) CSN6–CSN6 homodimer (PDBID 4E0Q) and (right) CSN5–CSN5 homodimer (PDBID 4F7O) complexes.

D) Ribbons comparison of CfBRCC36 in the CfBRCC36-KIAA0157ΔC complex with AMSH-LP bound to K63-linked di-Ub (PDBID 2ZNV). BRCC36 was superimposed on the AMSH-LP–di-Ub co-structure using the program Coot.

E) Zoom-in view of the diUb binding site of AMSH-LP and BRCC36.

F) Zoom-in view shows a 180° rotation of the view in **(E)**. Residues predicted to interact with distal and proximal ubiquitins and selected for mutational analysis are colored red. Ins-2 of AMSH-LP was removed for clarity.

Figure S5. Co-localization of BRCC36 and Abraxas with γH2AX

A) Quantification of foci numbers per cell +/– SD for BRCC36 mutants (left) and Abraxas mutants (right).

B) Dual color immunofluoresence using antibodies against HA and γH2AX was performed at 6 hours after 10 Gy IR in MEFs that stably expressed Flag-HA-Abraxas. Co-localization of WT Abraxas with γH2AX indicates successful recruitment to DNA double-strand breaks.

Figure S6. Structure of the pseudo DUB KIAA0157 and the CfBRCC36 QSQ active site mutant

A) Ribbons comparison of CfBRCC36 (blue) and CfKIAA0157 subunits (green) in the CfBRCC36-KIAA0157 complex. Zoom-in panel shows active site details. Superposition of subunits was performed using Coot.

B) Ribbons comparison of wild type CfBRCC36 (HSH) structure in blue with the CfBRCC36 His94Gln, Ser95Ser, His96Gln (QSQ) mutant in pink. Zoom-in panel shows active site details. The structure of the bound KIAA0157 subunit has been omitted for clarity.

Figure S7. Comparison of the inactive BRCC36 homodimer and the active BRCC36–KIAA0157 heterodimer

A) Stereo figure of a superimposed DrBRCC36 homodimer with a CfBRCC36–KIAA0157 heterodimer. E-loop (BRCC36) and the KIAA0157-specific E-loop supporting element are labeled. KIAA0157 possesses an insert (denoted the E-loop supporting element) not present in BRCC36 that buttresses a productive conformation of the E-loop of BRCC36.

B) Multiple sequence alignment (black = conserved, white = not conserved) of BRCC36 and KIAA0157 (KIAA) from the indicated species. Alignments were performed with MUSCLE and edited and displayed using ALINE. The secondary structure of BRCC36 (blue) and KIAA0157 (green) determined by DSSP is shown above and below the alignment. The ant BRCC36 and KIAA0157 sequences are numbered at the top and bottom of the alignment respectively. *Cf, C.*

floridanus; Hs, H. sapiens; Xl, X. laevis; Xt, X. tropicalis. The E-loop supporting element specific to KIAA0157 is shown in red box.

C) Conservation of the active site features of MPN^+ proteins. Superposition of MPN^+ domains of different JAMM/MPN domain proteins from various species. Conserved structural motifs and residues (shown as sticks) are labeled (top). Sequence comparison of MPN^+ domain proteins shown in top panel (bottom). Percent sequence identity of listed proteins to the ant BRCC36 is tabulated.

D) Coomassie-stained SDS-PAGE analysis of the indicated BRCC36–KIAA0157 mutant complexes analyzed in **Figure 5C**.

E) Size exclusion chromatography followed by multi angle light scattering (SEC-MALS) analysis of the indicated BRCC36–KIAA0157 mutant complexes analyzed in **Figure 5C**.

Figure S8. Validation of the higher-order packing arrangements of the BRCC36-KIAA0157ΔC heterodimer observed in the crystal environment

A) The centrally oriented BRCC36–KIAA0157 heterodimer (colored light blue and light green, respectively) forms major contacts (>700 $Å^2$) with four other heterodimers in the crystal lattice. The buried surface area for each contact is indicated. The BRCC36-KIAA0157ΔC heterodimer with the largest contact surface (super dimer 1) is colored dark blue and dark green respectively and others are colored black and grey respectively.

B) Refined *ab initio* bead model calculated by DAMMIN (top) and ribbons representation of structure of super dimer 1 shown in the same orientation (bottom).

C) Comparison of the measured scattering curve (open red circles) with the calculated scattering curve for the CfBRCC36–KIAA0157ΔC heterodimer (solid black lines). The chi value calculated from the CRYSOL fitting is indicated.

D) Comparison of the measured scattering curve (open red circles) with the calculated scattering curves for the four possible super dimers (super dimers 1 to 4) are shown from top to bottom with solid black lines. The chi values calculated from the CRYSOL fittings are indicated.

E) SEC-MALS analysis of WT CfBRCC36–KIAA0157ΔC complexes and the indicated super dimer mutants. Amount in percent represents the amount of peak 1 (dimer of dimers) as a fraction of the total input.

F) Summary of measured and calculated molecular weights for each peak of the indicated CfBRCC36–KIAA0157ΔC species.

G) Coomassie-stained SDS-PAGE analysis of the indicated CfBRCC36–KIAA0157ΔC complexes.

SUPPLEMENTAL TABLES

Table S1. Summary of genes referred to in this study and their accession numbers

Species	Full protein name	Gene name	NCBI/Uniprot ID
C. floridanus	CfBRCC36	*brcc3*	EFN61907.1
D. rerio	DrBRCC36	*brcc3*	XP_002666137.1
H. sapiens	HsBRCC36	*brcc3*	NP_001229569.1
C. floridanus	CfKIAA0157	*kiaa0157*	E2AB17
D. rerio	DrKIAA0157	*kiaa0157*	XP_005158832.1
H. sapiens	HsKIAA0157	*kiaa0157*	Q15018
C. floridanus	CfBRCC45	*Bre*	E2AN93
D. rerio	DrBRCC45	*Bre*	NP_001017777
H. sapiens	HsBRCC45	*Bre*	Q9NXR7
C. floridanus	CfMERIT40	*babam1*	E2ACB1
D. rerio	DrMERIT40	*babam1*	Q6AXK4
H. sapiens	HsMERIT40	*babam1*	Q9NWV8
H. sapiens	HsRAP80	*rap80*	Q96RL1
H. sapiens	HsSHMT2	*shmt2*	P34897

Table S2. Summary of mutated residues and their equivalent positions amongst different species

GENE	MUTATED RESIDUE	
	C. floridanus	***H. sapiens***
**BRCC36**	L20R	L23R
**BRCC36**	L24R	L27R
**BRCC36**	L20R + L24R	L23R + L27R
**BRCC36**	L170R	L229R
**BRCC36**	I217R	A278R
**BRCC36**	A205D	S266D
**BRCC36**	I212D	C273D
**BRCC36**	A205D + I212D	S266D + C273D
	C. floridanus	***H. sapiens***
**KIAA0157**	S16R	S11R
**KIAA0157**	L17R	A12R
**KIAA0157**	F20S	F15S
**KIAA0157**	S16R + L17R	S11R + A12R
**KIAA0157**	S16R + F20S	S11R + F15S
**KIAA0157**	L236R	V220R
**KIAA0157**	D247Y	E231Y
**KIAA0157**	V257Y	V241Y
**KIAA0157**	L236R + D247Y + V257Y	V220R + E231Y + V241Y
	***C. floridanus* KIAA0157**	***H. sapiens* Abraxas**
**ABRAXAS**	S16R	G16R
**ABRAXAS**	F20A	F20A
**ABRAXAS**	S16R + F20A	G16R + F20A
**ABRAXAS**	D247Y	E237Y
**ABRAXAS**	V250Y	V240Y

Table S3. Mutations of BRCC36, Abraxas and KIAA0157 genes found in cancer samples.

Source: COSMIC database, last accessed on 24 Novebmer 2014.

legend: CCHB = Coiled coil helical bundle
MPN = MPN domain
CTRR = C-terminal regualtory region deleted from KIAA0157 crystal construct

BRCC36 Mutations

Position	CDS Mutation	AA Mutation	Mutation ID (COSM)	Count	Type	Domain
10	c.28C>T	p.Q10*	756740	1	Substitution - Nonsense	MPN
11	c.32C>G	p.A11G	4137796	1	Substitution - Missense	MPN
12	c.34G>T	p.V12F	612023	1	Substitution - Missense	MPN
34	c.100G>A	p.V34I	69845	1	Substitution - Missense	MPN
44	c.130G>A	p.D44N	3559965	1	Substitution - Missense	MPN
55	c.163A>T	p.T55S	229646	1	Substitution - Missense	MPN
60	c.178C>T	p.R60C	1118318	1	Substitution - Missense	MPN
60	c.179G>A	p.R60H	1466965	1	Substitution - Missense	MPN
67	c.201T>A	p.D67E	457147	1	Substitution - Missense	MPN
69	c.205G>A	p.V69I	1118319	1	Substitution - Missense	MPN
76	c.227C>T	p.S76F	462275	1	Substitution - Missense	MPN
81	c.241C>T	p.R81*	210943	2	Substitution - Nonsense	MPN
82	c.245G>T	p.R82L	4154908	1	Substitution - Missense	MPN
90	c.269T>C	p.V90A	4108264	1	Substitution - Missense	MPN
129	c.385G>T	p.V129F	301775	1	Substitution - Missense	MPN
137	c.410G>A	p.R137H	457148	1	Substitution - Missense	MPN
167	c.499C>T	p.R167W	4137797	1	Substitution - Missense	MPN
178	c.532G>A	p.A178T	93984	1	Substitution - Missense	MPN
180	c.540G>T	p.K180N	1118321	1	Substitution - Missense	MPN
190	c.568G>T	p.D190Y	1118322	1	Substitution - Missense	MPN
211	c.631A>T	p.R211*	3844152	1	Substitution - Nonsense	MPN
213	c.637G>A	p.E213K	488185	1	Substitution - Missense	MPN
233	c.697G>T	p.V233L	403275	1	Substitution - Missense	CCHB
243	c.729G>C	p.E243D	3406178	1	Substitution - Missense	CCHB
258	c.772T>C	p.S258P	1466966	1	Substitution - Missense	CCHB
258	c.773C>G	p.S258*	50901	1	Substitution - Nonsense	CCHB
258	c.773C>T	p.S258L	1118323	1	Substitution - Missense	CCHB
270	c.810G>T	p.K270N	1118324	1	Substitution - Missense	CCHB
278	c.832G>A	p.A278T	1118325	1	Substitution - Missense	CCHB
282	c.844C>T	p.P282S	1118326	2	Substitution - Missense	CCHB
288	c.864G>C	p.E288D	306041	1	Substitution - Missense	CCHB
289	c.865delG	p.D289fs*27	4169833	1	Deletion - Frameshift	CCHB
291	c.871C>G	p.L291V	13845	4	Substitution - Missense	CCHB
291	c.872T>C	p.L291P	1466967	1	Substitution - Missense	CCHB
311	c.931G>T	p.E311*	1118327	1	Substitution - Nonsense	CCHB
312	c.935T>A	p.L312H	3424638	1	Substitution - Missense	CCHB
315	c.943C>G	p.L315V	3035068	1	Substitution - Missense	CCHB

Abraxas Mutations

Position	CDS Mutation	AA Mutation	Mutation ID (COSM)	Count	Type	Domain
9	c.25G>A	p.V9M	1431440	1	Substitution - Missense	MPN
34	c.100C>T	p.L34F	3826346	1	Substitution - Missense	MPN
39	c.115G>A	p.G39S	3661238	2	Substitution - Missense	MPN
39	c.115G>C	p.G39R	3661237	1	Substitution - Missense	MPN
42	c.126G>C	p.K42N	734439	1	Substitution - Missense	MPN
52	c.154G>A	p.D52N	3669307	1	Substitution - Missense	MPN
67	c.200G>T	p.C67F	1633819	1	Substitution - Missense	MPN
86	c.258G>T	p.K86N	275059	1	Substitution - Missense	MPN
93	c.279A>C	p.K93N	1058300	1	Substitution - Missense	MPN
105	c.313C>G	p.H105D	588729	1	Substitution - Missense	MPN
105	c.313C>T	p.H105Y	734440	1	Substitution - Missense	MPN
126	c.377C>T	p.S126L	211445	1	Substitution - Missense	MPN
136	c.407C>T	p.T136I	4126398	1	Substitution - Missense	MPN
142	c.424G>T	p.E142*	280964	1	Substitution - Nonsense	MPN
146	c.436A>G	p.T146A	1694626	1	Substitution - Missense	MPN
190	c.569G>A	p.G190D	1058299	1	Substitution - Missense	MPN
204	c.609_610insT	p.E204fs*1	300054	1	Insertion - Frameshift	CCHB
205	c.615_617delAG/	p.E205delE	1731484	1	Deletion - In frame	CCHB
232	c.694A>C	p.K232Q	4138530	1	Substitution - Missense	CCHB
234	c.700G>A	p.E234K	141087	1	Substitution - Missense	CCHB
252	c.754C>T	p.R252*	1058298	3	Substitution - Nonsense	CCHB
259	c.775G>T	p.G259*	1431437	1	Substitution - Nonsense	CCHB
304	c.910A>T	p.R304*	402488	1	Substitution - Nonsense	CTRR
310	c.929G>A	p.S310N	1727860	1	Substitution - Missense	CTRR
312	c.936C>A	p.N312K	4126397	1	Substitution - Missense	CTRR
318	c.952G>A	p.D318N	3940970	1	Substitution - Missense	CTRR
329	c.985C>A	p.H329N	588731	1	Substitution - Missense	CTRR
348	c.1042G>A	p.A348T	3760790	2	Substitution - Missense	CTRR
360	c.1079C>T	p.S360F	1058297	1	Substitution - Missense	CTRR
361	c.1081C>T	p.R361W	1694625	2	Substitution - Missense	CTRR
364	c.1091A>G	p.D364G	1058296	1	Substitution - Missense	CTRR
367	c.1099G>A	p.D367N	27186	1	Substitution - Missense	CTRR
367	c.1100A>G	p.D367G	1431435	3	Substitution - Missense	CTRR
378	c.1134C>A	p.N378K	3768035	1	Substitution - Missense	CTRR
409	c.1226T>G	p.F409C	1058295	1	Substitution - Missense	CTRR

KIAA0157 Mutations

Position	CDS Mutation	AA Mutation	Mutation ID (COSM)	Count	Type	
398	c.1192C>T	p.R398*	160936	1	Substitution - Nonsense	CTRR

SUPPLEMENTAL EXPERIMENTAL PROCEDURES

Cloning, protein expression and purification of CfBRCC36–KIAA0157ΔC complex

All proteins and complexes described here were produced using the Multibac baculovirus/insect cell system (ATG biosynthetics). Full-length CfBRCC36 (or mutants thereof) preceded by a 6-His purification tag and TEV cleavage sequence was cloned into a pACEBac-1 expression vector. Tag-less CfKIAA0157ΔC (or mutants thereof) was cloned in the same pACEBac-1 vector (containing 6-His-TEV-BRCC36) using the manufacturer's protocol. Bacmid DNA was generated in DH10MultiBac-Turbo cells (ATG Biosynthetics) following manufacturer's protocol, and virus amplification and cell expression in Sf9 cells was carried out using standard procedures. Cells were harvested by centrifugation for 15 min at 500 g and resuspended in ice-cold lysis buffer. Cells were lysed using an Avestin C3 cell disrupter (18,000 psi), and lysate was cleared by centrifugation at 30,000 g for 30 min and filtration using a 0.45 μm filter, before loading onto a 5 ml HiTrap IMAC HP column (GE Healthcare, USA). After washing with 10 column volumes (CV) of high salt wash buffer B, the complex was eluted with a gradient of 20-300 mM imidazole. Fractions containing the complex were pooled and 0.3-0.5 mg of His-tagged TEV protease was added prior to dialysis against 4 l of wash buffer A for 12-14 h. The TEV protease and non-cleaved His-CfBRCC36 were removed by subtraction on HiTrap IMAC resin. The cleaved protein was concentrated and resolved on a Sepharose-S200 16/60 column (GE Healthcare, USA), pre-equilibrated in 25 mM HEPES-Na pH 7.0, 150 mM NaCl and 5 mM DTT. Eluted peaks were analyzed by SDS-PAGE, and fractions containing >95% pure CfBRCC36–CfKIAA0157ΔC complex were combined, concentrated to 7-10 mg/ml, snap-frozen in liquid nitrogen and stored at –80 ^{o}C.

CfBRISCΔNΔC, DrBRISCΔNΔC, and the CfBRCC36-KIA0157ΔC–BRCC45 subcomplex, were cloned, expressed and purified as described for the CfBRCC36–KIAA0157ΔC subcomplex by exploiting a single poly-His tag fused to BRCC36. Cloning, protein expression and purification of HsBRISC, HsARISCΔN, and CfBRCC45–MERIT40ΔN, exploited the single poly-His tag fused to the BRCC45 subunit and were produced as described for the CfBRCC36–KIAA0157 complex.

Cloning, protein expression and purification of DrBRCC36 (residues 1–260) was essentially as described for CfBRCC36 above, without co-expression with KIAA0157.

Production of selenomethionine-substituted CfBRCC36–KIAA0157ΔC complex

Sf9 cells grown in suspension were first conditioned in methionine-free media by splitting 1:3 for two passages in ESF921ΔMet medium (Expression Systems, USA). The culture was split to 8×10^5 in methionine-free media and after 24 hours was infected with 1/15 of P2 virus. DL-selenomethionine was added 8 hours after infection at 100 mg/l, followed by a second addition at 24 hours. Cells were harvested 48 hours after infection and selenomethionine-substituted BRCC36–KIAA0157ΔC complex was purified as described above. Selenomethionine incorporation was verified (~60%) by mass spectrometry.

Protein crystallization, structure solution and refinement

WT CfBRCC36–CfKIAA0157ΔC complex was crystallized at 20 ^{o}C in sitting drops by mixing 0.75 μl of protein complex (5 mg/ml) with 0.75 μl of mother liquor consisting of 0.1 M Na HEPES (pH 7.0), 10% w/v PEG4000 and 10% (v/v) 2-propanol. Rectangular-shaped crystals (150 x 150 x 30 μm) appeared overnight and grew to full size over 72 h. Crystals were flash-frozen in liquid nitrogen using mother liquor supplemented with 30% glycerol as cryoprotectant. Data were collected at 100 K on station 24-ID-C, NE CAT beamline, Advanced Photon Source (APS) and processed using the XDS package (Kabsch, 2010) (see **Table 1** for data collection and refinement statistics). The structure was solved using the SHELX programing suite (Sheldrick, 2010). Phases were improved by density modification using Resolve (Adams et al., 2010), which also built an initial model improved further by Buccaneer (Cowtan, 2006). The structure was refined by iterative rounds of refinement using PHENIX (Adams et al., 2010) and manual model building with the program COOT (Emsley and Cowtan, 2004).

Mutant QSQ CfBRCC36–CfKIAA0157ΔC complex was crystallized at 4 ^{o}C in sitting drops by mixing 0.4 μl of 5 mg/ml protein complex and 1.5 molar excess K63-linked diUb with 0.4 μl of mother liquor consisting of 0.1 M KCl, 0.1 M HEPES-Na (pH 7.5) and 15% PEG6000. Rectangular-shaped crystals, similar to WT CfBRCC36–KIAA0157ΔC appeared overnight and grew to full size over 72 h.

DrBRCC36 was crystallized at 20 °C in sitting drops by mixing 0.4 μl of 2.5 mg/ml protein with 0.4 μl of mother liquor consisting of 0.8 M succinic acid (pH 7.0). Cube-shaped crystals appeared overnight and grew to full size (35 x 35 x 35 μm) over 48 h.

Small angle X-ray Scattering (SAXS) analysis

The CfBRCC36–KIAA0157ΔC complex was resolved over a Superdex-200 (GE Healthcare) size exclusion chromatography column and sample homogeneity was confirmed by dynamic light scattering. The X-ray scattering data were collected on a BioSAXS-1000 configured on the right port of a MicroMax-007HF X-ray generator using a 35 μl sample volume. Consecutive scans of 10, 15 and 60 minutes were collected at 10 °C for three different protein concentrations (3, 7.5 and 15 mg/ml). To generate the sample scattering curves, SAXSLab 3.0.0r1 software (Rigaku) was used to subtract buffer scattering from the protein scattering. SAXS profiles at the three concentrations were analyzed using the ATSAS program suite v.2.5.1 (Konarev et al., 2006). None of the samples showed interparticle interactions and they appeared to be folded as judged by the Kratky plot. The highest quality estimates determined from the Guinier plot and pair-distance distribution function were used to select the sample used for further processing (7.5 mg/ml and 60 minutes exposure time). The CfBRCC36–KIAA0157ΔC complex had a radius of gyration (Rg) of 40.8 ± 0.1 Å and Dmax of 138 Å using $0.008 < s < 0.3$ range. Ten *ab initio* models were generated using DAMMIF (Franke and Svergun, 2009) and averaged using DAMAVER (Volkov and Svergun, 2003). The model was refined with DAMMIN (Svergun, 1999) using the damstart.pdb file as the starting search volume. Theoretical scattering curves for the possible super dimers were generated and compared to the experimental scattering curves using CRYSOL (Svergun et al., 1995).

SEC–MALS analysis

BRCC36-containing complexes were analyzed using a SEC-MALS apparatus equipped with MiniDawn Treos and Optilab T-rEX detectors (Wyatt technologies, USA). Samples were run in a buffer containing 50 mM HEPES (pH 7.5), 150 mM NaCl and 1 mM TCEP and flow rate of 0.5 ml/min. *C. floridanus* proteins were run on a WTC015S5 (Wyatt technologies, USA) column at a concentration of 1 mg/ml. *D. rerio* and *H. sapiens* proteins were run on a WTC050S5 column (Wyatt technologies, USA) at the following concentrations: DrBRCC36, 0.8 mg/ml;

DrBRISCΔNΔC, 0.25 mg/ml; HsBRISCΔNΔC , 0.1 mg/ml and HsARISCΔN, 0.25 mg/ml. Data was processed using Astra 6.0.3.16 Software (Wyatt technologies, USA).

DUB activity assays

WT and mutant forms of BRCC36-containing complexes were assayed at 30 ^{o}C in DUB reaction buffer containing 50 mM HEPES-NaOH pH 7.0, 100 mM NaCl, 1 mg/ml BSA, 1 mM DTT and 0.03% Brij-35. Internally quenched fluorescent (IQF) K63-linked diUb (Lifesensors, catalog number: DU6303) was used as a reporter for DUB activity [1 part IQF diUB : 9 parts unlabeled K63-linked diUb prepared as described by Pickart and Raasi 2005 (Pickart and Raasi, 2005)]. 20-μl enzyme reactions were carried out in 384-well black flat-bottom low flange plates (Corning; 35373). Cleaved diUb was monitored by measuring fluorescence intensity (Ex 540 nm, Em. 580 nm) every 1 or 2 min over 20 min, and initial velocity (v_o) was calculated as the slope of the linear part of the reaction progress curve. Fluorescence intensity units were converted to nM product concentration using TAMRA-labeled ubiquitin (Lifesensors, catalog number: SI270T) as a standard. Enzyme concentrations for which a reasonable progress curve was obtained over 20 min were: 1 nM BRISC, 1 nM BRCC36–KIAA0157–BRCC45, 35-50 nM BRCC36–KIAA0157ΔC. Substrate concentration was 1 μM. At high enzyme concentrations, v_o was calculated over 3-5 min.

For data presented in **Fig. 6B**, v_o was measured at increasing diUb concentrations (0-12.5 μM) and enzymes were used at the following concentrations: 44 nM CfBRCC36 (WT)–KIAA0157ΔC and 35 nM CfBRCC36 (A205D)–KIAA0157ΔC, CfBRCC36 (I212D)–KIAA0157ΔC and CfBRCC36 (A205D+I212D)–KIAA0157ΔC. Kinetic rate constants and substrate inhibition (K_i) parameters were calculated using GraphPad Prism 5 and a modified Michaelis–Menten equation that takes into account substrate inhibition: $v_o = V_{max} \times [diUb] / (K_m + [diUb] \times (1 + [diUb] / K_i))$. To model substrate inhibition for A205D and I212D CfBRCC36 mutants, V_{max} was constrained to the calculated value from low substrate concentrations (up to 2.5 μM), for which the kinetics follow simple Michaelis–Menten behavior.

Native mass spectrometry measurements for HsBRISC

Full length HsBRISC was buffer exchanged sequentially into 100 mM aqueous ammonium acetate pH 6.8 buffer using centrifugal filter units with a cut-off of 50 kDa (Millipore, England). The HsBRISC concentration used for the mass spectrometry measurements was ~1 μM (assuming intact protein complexes). Instrumentation used and corresponding settings and procedures were described by (Koschubs et al., 2010).

Generation of fluorophore-conjugated K63-linked diUb

A K63-linked diUb chain with a reactive thiol group at the C-terminus of the proximal ubiquitin was generated using the procedure of Pickart and Raasi (Pickart and Raasi, 2005) with one modification. In place of ubiquitin D77, ubiquitin harboring an R75C mutation and lacking the last two glycine residues was used in the enzymatic reaction. 0.2 mM purified diUb chains were then labeled in 0.1 ml reaction volumes in buffer containing 25 mM HEPES-Na (pH 7.5), 100 mM NaCl and 0.5 mM TCEP, and stepwise addition of Alexa Fluor 488 C5 maleimide (Invitrogen) to a final 0.6 mM concentration. Reactions were carried out overnight in the dark at room temperature and were stopped by addition of 6 mM reduced glutathione. Free dye was removed using 2 ml Zebra™ desalting columns (ThermoFisher). Labeling efficiency was assessed by mass spectrometry at 64%.

Fluorescence polarization binding assays

Binding reactions were performed with 10 nM fluorophore-conjugated K63-linked diUb and the indicated protein concentrations in fluorescence polarization (FP) buffer consisting of 50 mM HEPES-NaOH pH 7.5, 100 mM NaCl, 1 mM TCEP, 0.03% Brij-35 and 1 mg/ml BSA. Binding reactions were equilibrated for 35 min in 20 μl reactions in 384-well black flat-bottom low flange plates (Corning, 3573). Fluorescence intensities were measured using a BioTek Synergy Neo plate reader and FP was calculated with the Gen5 Data Analysis Software using the formula: FP (in polarization units, P) = $(F_{parallel} - F_{perpendicular})/(F_{parallel} + F_{perpendicular})$. The instrument G factor and detection gains were auto scaled to a well containing tracer alone to correspond to a reading of 20 mP. K_d values were calculated by non-linear regression analysis of FP values performed in GraphPad Prism 5 using a one-site total binding model: $Y = B_{max}*X/(K_d+X) + NS*X + Background$ (NS = Slope of the nonlinear regression in Y units divided by X units; Background = Measured binding with no added ligand). For competition

binding assays the CfBRCC36–KIAA0157ΔC concentration was fixed at 10 μM (corresponding to ~80% of total binding signal) in the presence of varying concentrations of competitor. Competitor K48 linked diUb was generated as described by Pickart and Raasi (2005) using Lys48Arg donor ubiquitin and Gly75Stop acceptor ubiquitin. Data were fitted using the Prism built-in dose-response equation for inhibition with variable slope: $Y = Bottom+(Top—Bottom)/(1+10^{((LogIC_{50}—X)*h)})$.

Structure analysis and sequence alignments

Multiple sequence alignments were performed using MUSCLE (Edgar, 2004) and displayed, edited and annotated using ALINE (Bond and Schüttelkopf, 2009). Secondary structure was analyzed using DSSP (Kabsch and Sander, 1983) and buried surface area and residue contacts were calculated using the programs AREAIMOL and CONTACT from the CCP4 suite (Collaborative Computational Project, Number 4, 1994). Structure alignments were performed using Coot and structure representations were performed using The PyMOL Molecular Graphics System, Schrodinger, LLC.

Immunoprecipitation and Immunoblots

All immunoprecipitations were performed as previously described (Shao et al., 2009). Briefly, BRCC36 and KIAA0157 expressing cells were lysed in NETN 150 (0.5% NP40, 25 mM Tris pH 7.5, 150 mM NaCl, 0.5 mM EDTA with 1mM PMSF). Flag- IP was performed on lysate supernatants for 4 hours with agarose conjugated anti-Flag M2 beads (Sigma) prior to elution in 0.1 mM glycine pH 2.5. IP for Abraxas occurred under identical conditions except that lysis was performed in NETN buffer containing 300 mM NaCl. Immunoblots were performed with the following rabbit polyclonal antibodies at 1:1000 dilution that were generated to recombinant proteins: MERIT40, Abraxas, KIAA0157, BRCC36, RAP80, and BRCC45. Commercially available antibodies to BRCA1 (D9, Santa Cruz), HA epitope (HA.11 Covance) and SHMT2 (Cell Signaling) were used according to the manufacturer's directions.

Response to HSV infection

HSV lacking the ICP0 gene was used to infect cells at a multiplicity of infection of 0.1 for 2 hours. Media was then changed followed by a 5-hour incubation period. Cells were then lysed in

NETN150 and lysates were run on a 4-12% gradient SDS PAGE gel for immunoblot. Phospho-STAT1 and STAT1 antibodies were purchased from Cell Signaling Corporation and used according to the manufacturer's specifications.

Immunofluoresence of DNA damage foci

Cells were grown on coverslips and subsequently irradiated with 10 Gy IR using a Cs-137 irradiator. Cells were fixed 6 hours later using 3% paraformaldehyde, 2% sucrose as previously described by Shao et al, (Shao et al., 2009) and IF performed using antibodies to HA (HA.11, 1:1000 dilution) and γH2AX (rabbit polyclonal, Novus Biologics). Images were captured with a QImaging RETIGA-SRV camera connected to a Nikon Eclipse 80i miscroscope. All images for a given experiment were captured on the same day with the same exposure times in an 8-bit grayscale format. Images were analyzed using the ImageJ software (NIH). IR-induced foci were quantified by average foci numbers from 100 cells. Quantification was performed on greater than 100 cells in 3 independent experiments.

SUPPLEMENTAL REFERENCES

Adams, P.D., Afonine, P.V., Bunkoczi, G., Chen, V.B., Davis, I.W., Echols, N., Headd, J.J., Hung, L.W., Kapral, G.J., Grosse-Kunstleve, R.W., et al. (2010). PHENIX: a comprehensive Python-based system for macromolecular structure solution. Acta Crystallogr D Biol Crystallogr 66, 213–221.

Bond, C.S., and Schüttelkopf, A.W. (2009). ALINE: a WYSIWYG protein-sequence alignment editor for publication-quality alignments. Acta Crystallogr D Biol Crystallogr 65, 510–512.

Collaborative Computational Project, Number 4 (1994). The CCP4 suite: programs for protein crystallography. Acta Crystallogr D Biol Crystallogr 50, 760–763.

Cowtan, K. (2006). The Buccaneer software for automated model building. 1. Tracing protein chains. Acta Crystallogr D Biol Crystallogr 62, 1002–1011.

Edgar, R. (2004). MUSCLE: multiple sequence alignment with high accuracy and high throughput. Nucleic Acids Res.

Emsley, P., and Cowtan, K. (2004). Coot: model-building tools for molecular graphics. Acta Crystallogr D Biol Crystallogr 60, 2126–2132.

Franke, D., and Svergun, D.I. (2009). DAMMIF, a program for rapid ab-initio shape determination in small-angle scattering. J Appl Crystallogr 42, 342–346.

Kabsch, W. (2010). XDS. Urn:Issn:0907-4449 66, 125–132.

Kabsch, W., and Sander, C. (1983). Dictionary of protein secondary structure: pattern recognition of hydrogen-bonded and geometrical features. Biopolymers 22, 2577–2637.

Konarev, P.V., Petoukhov, M.V., Volkov, V.V., and Svergun, D.I. (2006). ATSAS 2.1, a program package for small-angle scattering data analysis. J Appl Crystallogr 39, 277–286.

Koschubs, T., Lorenzen, K., Baumli, S., Sandström, S., Heck, A.J.R., and Cramer, P. (2010). Preparation and topology of the Mediator middle module. Nucleic Acids Res. 38, 3186–3195.

Pickart, C.M., and Raasi, S. (2005). Methods in Enzymology (Elsevier).

Shao, G., Patterson-Fortin, J., Messick, T.E., Feng, D., Shanbhag, N., Wang, Y., and Greenberg, R.A. (2009). MERIT40 controls BRCA1-Rap80 complex integrity and recruitment to DNA double-strand breaks. Genes Dev 23, 740–754.

Sheldrick, G.M. (2010). Experimental phasing with SHELXC/D/E: combining chain tracing with density modification. Acta Crystallogr D Biol Crystallogr 66, 479–485.

Svergun, D.I. (1999). Restoring low resolution structure of biological macromolecules from

solution scattering using simulated annealing. Biophys J 76, 2879–2886.

Svergun, D., Barberato, C., and Koch, M.H.J. (1995). CRYSOL – a Program to Evaluate X-ray Solution Scattering of Biological Macromolecules from Atomic Coordinates

. J Appl Crystallogr 28, 768–773.

Volkov, V.V., and Svergun, D.I. (2003). Uniqueness of ab initio shape determination in small-angle scattering. Journal of Applied Crystallography.
